# A Path Toward Explainable AI and Autonomous Adaptive Intelligence: Deep Learning, Adaptive Resonance, and Models of Perception, Emotion, and Action

**DOI:** 10.3389/fnbot.2020.00036

**Published:** 2020-06-25

**Authors:** Stephen Grossberg

**Affiliations:** Graduate Program in Cognitive and Neural Systems, Departments of Mathematics & Statistics, Psychological & Brain Sciences, and Biomedical Engineering, Center for Adaptive Systems, Boston University, Boston, MA, United States

**Keywords:** Adaptive Resonance Theory, deep learning, explainable AI, visual boundaries and surfaces, category learning, emotion, consciousness, arm and speech movement

## Abstract

Biological neural network models whereby brains make minds help to understand autonomous adaptive intelligence. This article summarizes why the dynamics and emergent properties of such models for perception, cognition, emotion, and action are explainable, and thus amenable to being confidently implemented in large-scale applications. Key to their explainability is how these models combine fast activations, or short-term memory (STM) traces, and learned weights, or long-term memory (LTM) traces. Visual and auditory perceptual models have explainable conscious STM representations of visual surfaces and auditory streams in surface-shroud resonances and stream-shroud resonances, respectively. Deep Learning is often used to classify data. However, Deep Learning can experience catastrophic forgetting: At any stage of learning, an unpredictable part of its memory can collapse. Even if it makes some accurate classifications, they are not explainable and thus cannot be used with confidence. Deep Learning shares these problems with the back propagation algorithm, whose computational problems due to non-local weight transport during mismatch learning were described in the 1980s. Deep Learning became popular after very fast computers and huge online databases became available that enabled new applications despite these problems. Adaptive Resonance Theory, or ART, algorithms overcome the computational problems of back propagation and Deep Learning. ART is a self-organizing production system that incrementally learns, using arbitrary combinations of unsupervised and supervised learning and only locally computable quantities, to rapidly classify large non-stationary databases without experiencing catastrophic forgetting. ART classifications and predictions are explainable using the attended critical feature patterns in STM on which they build. The LTM adaptive weights of the fuzzy ARTMAP algorithm induce fuzzy IF-THEN rules that explain what feature combinations predict successful outcomes. ART has been successfully used in multiple large-scale real world applications, including remote sensing, medical database prediction, and social media data clustering. Also explainable are the MOTIVATOR model of reinforcement learning and cognitive-emotional interactions, and the VITE, DIRECT, DIVA, and SOVEREIGN models for reaching, speech production, spatial navigation, and autonomous adaptive intelligence. These biological models exemplify complementary computing, and use local laws for match learning and mismatch learning that avoid the problems of Deep Learning.

## 1. Toward Explainable Ai and Autonomous Adaptive Intelligence

### 1.1. Foundational Problems With Back Propagation and Deep Learning

This *Frontiers* Research Topic about Explainable Artificial Intelligence aims to clarify some fundamental issues concerning biological and artificial intelligence. As its Abstract summarizes: “Though Deep Learning is the main pillar of current AI techniques and is ubiquitous in basic science and real-world applications, it is also flagged by AI researchers for its black-box problem: it is easy to fool, and it also cannot explain how it makes a prediction or decision.”

The *Frontiers* Research Topic Abstract goes on to summarize the kinds of real world situations in which a successful adaptive classification algorithm must be able to learn: “In both … biological brains and AI, intelligence involves decision-making using data that are noisy and often ambiguously labeled. Input data can also be incorrect due to faulty sensors. Moreover, during the skill acquisition process, failure is required to learn.”

Deep Learning uses the back propagation algorithm to learn how to predict output vectors in response to input vectors. These models are based upon the Perceptron learning principles introduced by Rosenblatt (1958, 1962), who also introduced the term “back propagation.” Back propagation was developed between the 1970s and 1980s by people like Amari (1972), Werbos (1974, 1994), and Parker (1985, 1986, 1987), reaching its modern form and being successfully simulated in applications by Werbos (1974). The algorithm was then popularized in 1986 by an article of Rumelhart et al. (1986). Schmidhuber (2020) provides a detailed historical account of many additional scientists who contributed to this development.

Both back propagation and Deep Learning are typically defined by a feedforward network whose adaptive weights can be altered when, in response to an input vector, its adaptive filter generates an output vector that mismatches the correct, or desired, output vector. “Failure is required to learn” in back propagation by computing a scalar error signal that calibrates the distance between the actual and desired output vectors. Thus, at least in the algorithm's classical form, learning is *supervised* and requires that a desired output vector be supplied by a teacher on every learning trial, so that the error signal between the actual and desired output vectors can be computed.

**Figure 1:**
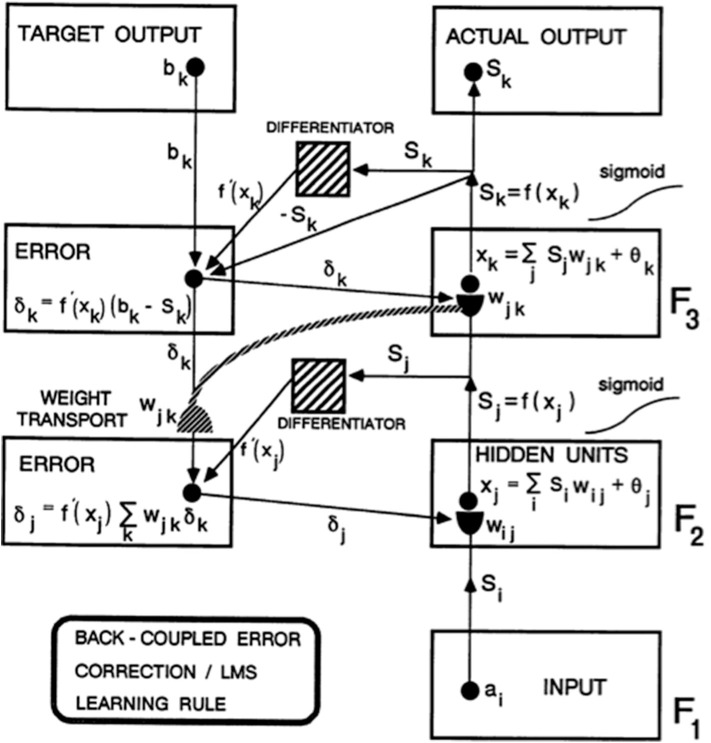
Circuit diagram of the back propagation model. Input vector a_i_ in level F_1_ sends a sigmoid signal S_i_ = f(a_i_) that is multiplied by learned weights w_ij_ on their way to level F_2_. These LTM-weighted signals are added together at F_2_ with a bias term θ_j_ to define x_j_. A sigmoid signal S_j_ = f(x_j_) then generates outputs from F_2_ that activate two pathways. One pathway inputs to a Differentiator. The other pathway gets multiplied by adaptive weight w_jk_ on the way to level F_3_. At level F_3_, the weighted signals are added together with a bias term θ_k_ to define x_k_. A sigmoid signal S_k_ = f(x_k_) from F_3_ defines the Actual Output of the system. This Actual Output S_k_ is subtracted from a Target Output b_k_ via a back-coupled error correction step. The difference b_k_ – S_k_ is also multiplied by the term f′(x_k_) that is computed at the Differentiator from level F_3_. One function of the Differentiator step is to ensure that the activities and weights remain in a bounded range, because if x_k_ grows too large, then f′(x_k_) approaches zero. The net effect of these operations is to compute the Error δ_k_ = f′(x_k_)(b_k_ – S_k_) that sends a top-down output signal to the level just below it. On the way, each δ_k_ is multiplied by the bottom-up learned weights w_jk_ at F_3_. These weights reach the pathways that carry δ_k_ via the process of *weight transport*. Weight transport is clearly a non-local operation relative to the network connections that carry locally computed signals. All the δ_k_ are multiplied by the transported weights w_jk_ and added. This sum is multiplied by another Differentiator term f′(x_i_) from level F_2_ to keep the resultant product δ_j_ bounded. δ_j_ is then back-coupled to adjust all the weights w_ij_ in pathways from level F_1_ to F_2_ [figure reprinted and text adapted with permission from Carpenter (1989)].

As Figure 1 from Carpenter (1989) explains in greater detail, this error signal back-propagates to the pathway endings where the adaptive weights occur in the algorithm, and alters them to reduce the error. The location in the algorithm where the error signal is computed is not where the adaptive weights are computed at the ends of pathways within the adaptive filter. *Weight transport* of the error signal across the network is thus needed to train the adaptive weights (Figure 1). This transport is “non-local” in the sense that there are no pathways from where the error signal is computed along which it can naturally flow to where the adaptive weights are computed. *Slow learning* occurs in the sense that adaptive weights change only slightly on each learning trial to gradually reduce the error signals. *Fast learning*, that would zero the error signal after any single erroneous prediction, could destabilize learning and memory because the new prediction that is being learned could massively recode the information that was previously learned, notwithstanding its continued correctness in the original environment.

This kind of error-based supervised learning has other foundational computational problems that were already known in the 1980s. One of the most consequential ones is that, even if learning is slow, Deep Learning can experience *catastrophic forgetting* (McCloskey and Cohen, 1989; Ratcliff, 1990; French, 1999): During any trial during learning of a large database, an unpredictable part of its memory can suddenly collapse. French (1999) traces these problems to the fact that all inputs are processed through a shared set of learned weights, and the absence of a mechanism within the algorithm to selectively buffer previous learning that is still predictively useful. Catastrophic forgetting can, in fact, occur in any learning algorithm whose shared weight updates are based on the gradient of the error in response to the current batch of data points, while ignoring past batches.

In addition, in the back propagation and Deep Learning algorithms, there are no perceptual, cognitive, emotional, or motor representations of the fast information processing that biological brains carry out, and no way for the algorithm to pay attention to information that may be predictively important in one environment, but irrelevant in another. The only residue of previous experiences lies in the changes that they caused in the algorithm's adaptive weights (within the hemidisks in Figure 1). All future experiences are non-selectively filtered through this shared set of weights.

French (1999) also reviews multiple algorithmic refinements that were made, and continue to be made, to at least partially overcome these problems. The main computational reality is, however, that such efforts are reminiscent of the epicycles that were added to the Ptolemaic model of the solar system to make it work better. The need for epicycles was obviated by the Copernican model.

Multiple articles have been written since French (1999) in an effort to overcome the catastrophic forgetting problem. The article by Kirkpatrick et al. (2017) is illustrative. These authors “overcome this limitation and train networks that can maintain expertise on tasks that they have not experienced for a long time … by selectively slowing down learning on the weights important for those tasks … in supervised learning and reinforcement learning problems” (p. 3521).

The method that is used to carry out this process requires extensive external supervision, uses non-locally computed mathematical quantities and equations that implement a form of batch learning, and operates off-line. In particular, to determine “which weights are most important for a task,” this method proceeds by “optimizing the parameters [by] finding their most probable values given some data *D*” by computing the “conditional probability from the prior probability of the parameters *p*(θ) and the probability of the data *p*(*D*/θ) by using Bayes' rule” (p. 3522). Computing these probabilities requires non-local, simultaneous, or batch, access to multiple learning trials by an omniscient observer who can compute the afore-mentioned probabilities off-line.

Further external manipulation is needed because “The true posterior probability is intractable so…we approximate the posterior as a Gaussian distribution with mean given by the parameters $\theta_{Z}^{*}$ and a diagonal precision given by the diagonal of the Fisher information matrix F” (p. 3522). This approximation leads to the problem of minimizing a functional that includes a parameter **λ** that “sets how important the old task is compared with the new one” (p. 3522).

Related approaches to evaluating the importance of a learned connection, or its “connection cost,” include evolutionary algorithms that compute an evolutionary cost for each connection (e.g., Clune et al., 2013). Evolutionary algorithms are inspired by Darwinian evolution. They include a mechanism to search through various neural network configurations for the best weights whereby the model can solve a problem (Yao, 1999). Catastrophic forgetting in this setting is ameliorated by learning weights within one module without engaging other parts of the network. This approach experiences the same kinds of conceptual problems that Kirkpatrick et al. (2017) does.

Another way to create different modules for different tasks is to restrict task-specific learning in a local group of network nodes and connections by using diffusion-based neuromodulation. This method places “point sources at specific locations within an ANN that emit diffusing learning signals that correspond to the positive and negative feedback for the tasks being learned” (Velez and Clune, 2017). In addition to conceptual problems about how these locations are chosen to try to overcome the catastrophic forgetting that obtains without diffusion, this algorithm seems thus far to have only been applied to a simple foraging task whose goal is “to learn which food items are nutritious and should be eaten, and which are poisonous and should not be eaten” across seasons where the nutritional value of the food items may change.

A related problem is solved by the ARTMAP neural network that is discussed below (Carpenter et al., 1991) when it learns to distinguish highly similar edible and poisonous mushrooms (Lincoff, 1981) with high predictive accuracy, and does so without experiencing catastrophic forgetting or using neuromodulation. ARTMAP has also successfully classified much larger databases, such as the Boeing design retrieval system that is listed below.

Although these model modifications and variations may at least partially ameliorate the catastrophic forgetting problem, I consider them to be epicycles because they attempt to overcome fundamental problems of the models' core learning properties.

Another core problem of both back propagation and Deep Learning, which no number of epicycles can totally cure, is thus that they do not solve what I have called the *stability-plasticity dilemma*; that is, the ability to learn quickly (plasticity) without experiencing catastrophic forgetting (stability) over the lifetime of a human or machine. Deep Learning is, in this sense, unreliable. When the stability-plasticity dilemma is overcome in a principled way, epicycles become unnecessary and reliable results are obtained, as I will show below. In particular, the biological learning models, such as ARTMAP that will be described below function in an autonomous or self-organizing way, use only locally computable quantities, and can incrementally learn on-line and in real time. When their learning is supervised, the teaching signals occur naturally in the environments within which the learning occurs.

Other computational problems than the tendency for catastrophic forgetting occur in multi-layer Perceptrons like back propagation and Deep Learning. Even when accurate predictions are made to some data, the basis upon which these predictions are made is unknown in both back propagation and Deep Learning. As noted above, “it is … flagged by AI researchers for its black-box problem: it is easy to fool, and … cannot explain how it makes a prediction or decision.” Deep Learning thus does not solve the *Explainable AI Problem* (https://www.darpa.mil/attachments/XAIProgramUpdate.pdf). Its predictions cannot be trusted. It is also not known whether predictions about related data will be correct or incorrect. Urgent life decisions, including medical and financial ones, cannot confidently use an algorithm with these weaknesses.

The popularity of back propagation decreased as the above kinds of problems became increasingly evident during the 1980s. Deep Learning recently became popular again after the worst effects of slow and unstable learning were overcome by the advent of very fast computers and huge online databases (e.g., millions of pictures of cats), at least when these databases are presented without significant statistical biases. Although slow learning still requires many learning trials, very fast computers enable large numbers of trials to learn from many exemplars and thereby at least partially overcome the memory instabilities that can occur in response to biased small samples (Hinton et al., 2012; Le Cun et al., 2015). With these problems partially ameliorated, albeit not solved, many kinds of practitioners, including large companies like Apple and Google, have been able to use Deep Learning in new applications despite its foundational problems.

### 1.2. “Throw It All Away and Start Over”?

It is perhaps because the core problems of these algorithms have not been solved that Geoffrey Hinton, who played a key role in developing both back propagation and Deep Learning, said in an *Axios* interview on September 15, 2017 (Le Vine, 2017) that he is “deeply suspicious of back propagation … I don't think it's how the brain works. We clearly don't need all the *labeled data* … My view is, *throw it all away and start over*” (italics mine).

The remainder of the article demonstrates that the problems of back propagation and Deep Learning that led Hinton to the conclusions in his Axios interview have been solved. These solutions are embodied in explainable neural network models that were discovered by analyzing how human and other advanced brains realize autonomous adaptive intelligence. In addition to explaining and predicting many psychological and neurobiological data, these models have been used to solve outstanding problems in engineering and technology. Section 2 summarizes how explainable cognitive processes use Adaptive Resonance Theory, or ART, circuits to learn to attend, recognize, and predict objects and events in environments whose statistical properties can rapidly and unexpectedly change. Section 3 summarizes explainable models of biological vision and audition, such as the FACADE model of 3D vision and figure-ground perception, which propose how resonant dynamics support conscious perception and recognition of visual and auditory qualia. Section 4 describes explainable models of cognitive-emotional interactions, such as the MOTIVATOR model, whose processes of reinforcement learning and incentive motivational learning enable attention to focus on valued goals and to release actions aimed at acquiring them. Section 5 summarizes explainable motor models, such as the DIRECT and DIVA models, that can learn to control motor-equivalent reaching and speaking behaviors. Section 6 combines these models with the GridPlaceMap model of spatial navigation into the SOVEREIGN neural architecture that provides a unified foundation for autonomous adaptive intelligence of a mobile agent. Section 7 provides a brief conclusion. The functional dynamics of multiple brain processes are clarified by these models, as are unifying computational principles, such as Complementary Computing and the use of Difference Vectors to control reaching, speaking, and navigation.

## 2. Adaptive Resonance Theory

### 2.1. Use Adaptive Resonance Theory Instead: ART as a Computational and Biological Theory

As I noted above, the problems of back propagation have been well-known since the 1980s. An article that I published in 1988 (Grossberg, 1988) listed 17 differences between back propagation and the biologically-inspired Adaptive Resonance Theory, or ART, that I introduced in 1976 and that has been steadily developed by many researchers since then, notably Gail Carpenter. These differences can be summarized by the following bullets:

- Real-time (on-line) learning vs. lab-time (off-line) learning
- Learning in non-stationary unexpected world vs. in stationary controlled world
- Self-organized unsupervised or supervised learning vs. supervised learning
- Dynamically self-stabilize learning to arbitrarily many inputs vs. catastrophic forgetting
- Maintain plasticity forever vs. externally shut off learning when database gets too large
- Effective learning of arbitrary databases vs. statistical restrictions on learnable data
- Learn internal expectations vs. impose external cost functions
- Actively focus attention to selectively learn critical features vs. passive weight change
- Closing vs. opening the feedback loop between fast signaling and slower learning
- Top-down priming and selective processing vs. activation of all memory resources
- Match learning vs. mismatch learning: Avoiding the noise catastrophe
- Fast and slow learning vs. only slow learning: Avoiding the oscillation catastrophe
- Learning guided by hypothesis testing and memory search vs. passive weight change
- Direct access to globally best match vs. local minima
- Asynchronous learning vs. fixed duration learning: A cost of unstable slow learning
- Autonomous vigilance control vs. unchanging sensitivity during learning
- General-purpose self-organizing production system vs. passive adaptive filter.

This list summarizes ART properties that overcome all 17 of the computational problems of back propagation and Deep Learning. Of particular relevance to the above discussion is the third of the 17 differences between back propagation and ART; namely, that ART does not need labeled data to learn.

ART exists in two forms: as algorithms that are designed for use in large-scale applications to engineering and technology, and as an incrementally developing biological theory. In its latter form, ART is now the most advanced cognitive and neural theory about how our brains learn to attend, recognize, and predict objects and events in a rapidly changing world that can include many unexpected events. As of this writing, ART has explained and predicted more psychological and neurobiological data than other available theories about these processes, and all of the foundational ART hypotheses have been supported by subsequent psychological and neurobiological data. See Grossberg (2013, 2017a,b); Grossberg (2018, 2019b) for reviews that support this claim and refer to related articles that have explained and predicted much more data since 1976 than these reviews can.

### 2.2. Deriving ART From a Universal Problem in Error Correction Clarifies Its Range of Applications

ART circuit designs can be derived from a thought, or Gedanken, experiment (Grossberg, 1980) that does not require any scientific knowledge to carry out. This thought experiment asks the question: How can a coding error be corrected if no individual cell knows that one has occurred? As Grossberg (1980, p. 7) notes: “The importance of this issue becomes clear when we realize that erroneous cues can accidentally be incorporated into a code when our interactions with the environment are simple and will only become evident when our environmental expectations become more demanding. Even if our code perfectly matched a given environment, we would certainly make errors as the environment itself fluctuates.”

The answers to this purely logical inquiry about error correction are translated at every step of the thought experiment into processes operating autonomously in real time with only locally computed quantities. The power of such a thought experiment is to show how, when familiar environmental constraints on incremental knowledge discovery are overcome in a self-organizing manner, then ART circuits naturally emerge. This fact suggests that ART designs may, in some form, be embodied in all future autonomous adaptive intelligent devices, whether biological or artificial.

Perhaps this is why ART has done well in benchmark studies where it has been compared with other algorithms, and has been used in many large-scale engineering and technological applications, including engineering design retrieval systems that include millions of parts defined by high-dimensional feature vectors, and that were used to design the Boeing 777 (Escobedo et al., 1993; Caudell et al., 1994). Other applications include classification and prediction of sonar and radar signals, of medical, satellite, face imagery, social media data, and of musical scores; control of mobile robots and nuclear power plants, cancer diagnosis, air quality monitoring, strength prediction for concrete mixes, solar hot water system monitoring, chemical process monitoring, signature verification, electric load forecasting, tool failure monitoring, fault diagnosis of pneumatic systems, chemical analysis from ultraviolent and infrared spectra, decision support for situation awareness, vision-based driver assistance, user profiles for personalized information dissemination, frequency-selective surface design for electromagnetic system devices, Chinese text categorization, semiconductor manufacturing, gene expression analysis, sleep apnea and narcolepsy detection, stock association discovery, viability of recommender systems, power transmission line fault diagnosis, million city traveling salesman problem, identification of long-range aerosol transport patterns, product redesign based on customer requirements, photometric clustering of regenerated plants of gladiolus, manufacturing cell formation with production data, and discovery of hierarchical thematic structure in text collections, among others. References and discussion of these and other applications and their biological foundations are found in Grossberg (2020).

As a result of these successes, ART has become one of the standard neural network models to which practitioners turn to solve their applications. See the web site http://techlab.bu.edu/resources/articles/C5 of the CNS Tech Lab for a partial list of illustrative benchmark studies and technology transfers. Readers who would like a recent summary of the many applications of ART to large-scale applications in engineering may want to look at the December, 2019, issue of the journal *Neural Networks*. The following two articles by Da Silva et al. (2019) and Wunsch (2019) from that special issue are of particular interest in this regard: https://arxiv.org/pdf/1910.13351.pdf and https://arxiv.org/pdf/1905.11437.pdf.

### 2.3. ART Is an Explainable Self-Organizing Production System in a Non-stationary World

ART is more than a feedforward adaptive filter. Although “during the skill acquisition process, failure is required to learn” in any competent learning system, ART goes beyond the kind of learning that is due just to slow modifications of adaptive weights in a feedforward filter. Instead, ART is a *self-organizing production system* that can incrementally learn, during unsupervised and supervised learning trials, to rapidly classify arbitrary non-stationary databases without experiencing catastrophic forgetting. In particular, ART can learn an entire database using fast learning on a single learning trial (e.g., Carpenter and Grossberg, 1987, 1988).

ART's predictions are explainable using both its activity patterns, or short-term memory (STM) traces, and its adaptive weights, or long-term memory (LTM) traces. I will summarize more completely below why both the STM and LTM traces in ART systems are explainable. For example, at any stage of learning, adaptive weights of the fuzzy ARTMAP algorithm can be translated into fuzzy IF-THEN rules that explain what combinations of features, and within what range, together predict successful outcomes (Carpenter et al., 1992). In every ART model, due to matching of bottom-up feature patterns with learned top-down expectations, an attentional focus emerges that selects the activity patterns of *critical features* that are deemed to be predictively important based on past learning. Already learned critical feature patterns are refined, and new ones discovered, to be incorporated through learning in the recognition categories that control model predictions. Such explainable STM and LTM properties are among the reasons that ART algorithms can be used with confidence to help solve large-scale real world problems.

### 2.4. Competition, Learned Expectation, and Attention

ART's good properties depend critically upon the fact that it supplements its feedforward, or *bottom-up*, adaptive filter circuits with two types of feedback interactions. The first type of feedback occurs in recurrent competitive, or lateral inhibitory, interactions at each processing stage. These competitive interactions normalize activity patterns, a property that is often called *contrast normalization* (Grossberg, 1973, 1980; Heeger, 1992). At the level of feature processing, they help to choose the critical features. At the level of category learning, they help to choose the contextually most favored recognition categories. Such competitive interactions do not exist in back propagation or Deep Learning.

**Figure 2:**
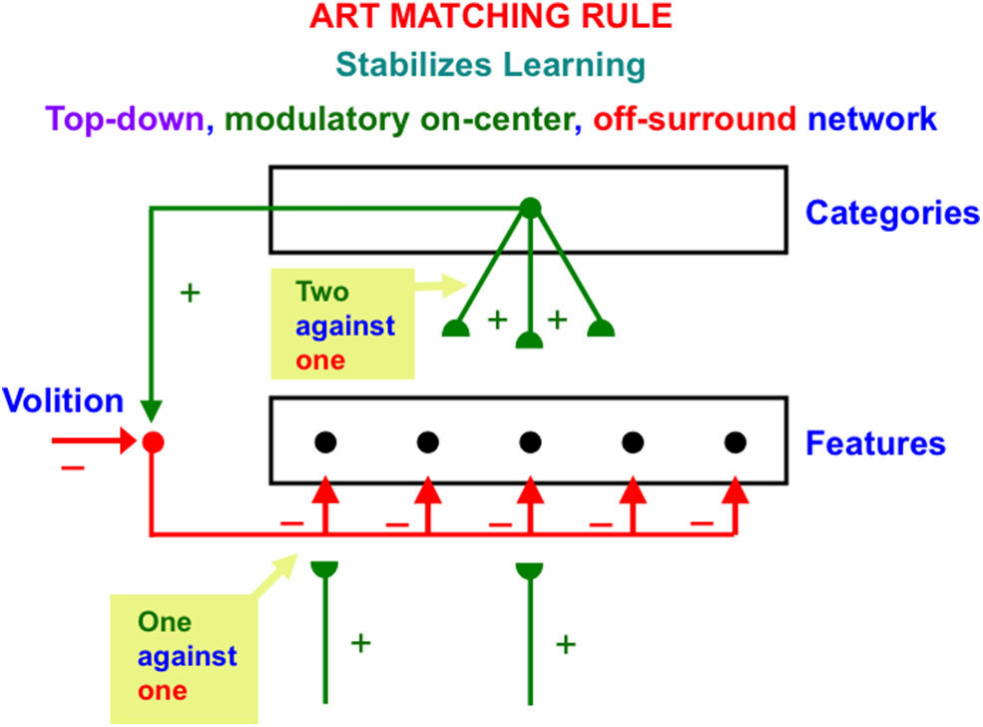
The ART Matching Rule circuit enables bottom-up inputs to fire their target cells, top-down expectations to provide excitatory modulation of cells in their on-center while inhibiting cells in their off-surround, and a convergence of bottom-up and top-down signals to generate an attentional focus at matched cells while continuing to inhibit unmatched cells in the off-surround [adapted with permission from Grossberg (2017b)].

ART also includes learned top-down expectations that are matched against bottom-up input patterns to focus attention using a type of circuit that obeys the ART Matching Rule. The top-down pathways that realize the ART Matching Rule form a *modulatory on-center, off-surround network* (Figure 2). The off-surround network includes the competitive interactions that were mentioned in the last paragraph. This network realizes the following properties:

When a bottom-up input pattern is received at a processing stage, it can activate its target cells if no other inputs are received. When a top-down expectation is the only active input source, it can provide excitatory modulatory, or priming, signals to cells in its on-center, and driving inhibitory signals to cells in its off-surround. The on-center is modulatory because the off-surround also inhibits the on-center cells, and these two input sources are approximately balanced (Figure 2). When a bottom-up input pattern and a top-down expectation are both active, cells that receive both bottom-up excitatory inputs and top-down excitatory priming signals can fire (“two-against-one”), while other cells in the off-surround are inhibited, even if they receive a bottom-up input (“one-against-one”). In this way, the only cells that fire are those whose features are “expected” by the top-down expectation. An attentional focus then starts to form at these cells.

ART learns how to focus attention upon the *critical feature patterns* that are selected during sequences of learning trials with multiple bottom-up inputs, while suppressing irrelevant features and noise. The critical features are the ones that contribute to accurate predictions as learning proceeds. This attentional selection process is one of the ways that ART successfully overcomes problems noted in the description of this *Frontiers* special topic by managing “data that are noisy and often ambiguously labeled,” as well as data that “can also be incorrect due to faulty sensors.” Only reliably predictive feature combinations will eventually control ART decisions via its ability to pay attention to task-relevant information.

Back propagation and Deep Learning do not compute STM activation patterns, learned LTM top-down expectations, or attended STM patterns of critical features. Because back propagation and Deep Learning are just feedforward adaptive filters, they do not do any fast information processing using STM patterns, let alone attentive information processing that can selectively use critical features.

### 2.5. Self-Organizing Production System: Complementary Computing

Because of this limitation, back propagation and Deep Learning can only correct an error using labeled data in which the output vector that embodies an incorrect prediction is mismatched with the correct prediction, thereby computing an error signal that uses weight transport to non-locally modify the adaptive weights that led to the incorrect prediction (Figure 1).

**Figure 3:**
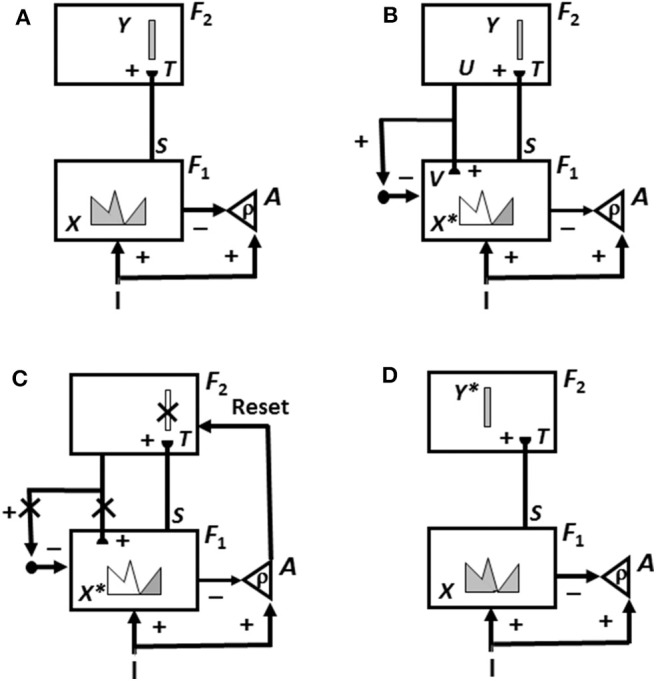
The ART hypothesis testing and learning cycle whereby bottom-up input patterns that are sufficiently mismatched by their top-down expectations can drive hypothesis testing and memory search leading to discovery of recognition categories that can match the bottom-up input pattern well-enough to trigger resonance and learning. See the text for details [adapted with permission from Carpenter and Grossberg (1988)].

This is not the case in either advanced brains or the biological neural networks like ART that model them. In ART, an unexpected outcome can be caused either by a mismatch of the predicted outcome with what actually occurs, or merely by the *unexpected non-occurrence* of the predicted outcome. In either situation, a mismatch causes a burst of *non-specific arousal* that calibrates how unexpected, or novel, the outcome is. This arousal burst can initiate *hypothesis testing* and *memory search*, which automatically leads to the discovery and choice of a better recognition category upon which to base predictions in the future. Figure 3 and its caption detail how an ART system can carry out hypothesis testing to discover and learn a recognition category whereby to better represent a novel situation.

When a new, but familiar, input is presented, it too triggers a memory search to activate the category that codes it. This is typically a very short cycle of just one reset event before the globally best matching category is chosen.

The combination of learned recognition categories and expectations, novelty responses, memory searches by hypothesis testing, and the discovery of rules are core processes in ART that qualify it as a self-organizing production system. These processes have been part of ART since it was introduced in 1976 (Grossberg, 1976a,b, 1978, 1980, 2017b).

Why does ART need both an *attentional system* in which learning and recognition occur (levels *F*_1_ and *F*_2_ in Figure 3), and an orienting system (*A* in Figure 3) that drives memory search and hypothesis testing by the attentional system until a better matching, or entirely new, category is found there? This design enables ART to solve a problem that occurs in a system that learns only when a good enough match occurs. How is anything new ever learned in a match learning system? The attentional and orienting systems have computationally *complementary* properties that solve this problem: A sufficiently bad mismatch between an active top-down expectation and a bottom-up input pattern, say in response to a novel input, can drive a memory search that continues until the system discovers a new approximate match, which can either refine learning of an old category or begin learning of a new one.

As described more fully in Grossberg (2017b), the complementary properties of the attentional and orienting systems are as follows: The attentional system supports *top-down, conditionable, specific*, and *match* properties that occur during an attentive match, whereas an orienting system mismatch triggers *bottom-up, unconditionable, non-specific*, and *mismatch* properties (Figure 3C). This is just one example of the general design principle of *complementary computing* that organizes many brain processes. See Grossberg (2000, 2017b) for more examples.

The ART *attentional system* for visual category learning includes brain regions, such as prestriate visual cortex, inferotemporal cortex, and prefrontal cortex, whereas the ART orienting system includes the non-specific thalamus and the hippocampal system. See Carpenter and Grossberg (1993) and Grossberg and Versace (2008) for relevant data.

### 2.6. ART Search and Learning Cycle to Discover Attended Critical Feature Patterns

The ART hypothesis testing and learning cycle (see Figure 3) explains how ART searches for and learns new recognition categories using cycles of match-induced resonance and mismatch-induced reset. These recognition categories are learned from the critical features that ART attends as a result of this hypothesis testing cycle, which proceeds as follows:

First, as in Figure 3A, an input pattern *I* activates feature detectors at level *F*_1_, thereby creating an activity pattern *X*. Pattern *X* is drawn as a continuous pattern that interpolates the activities at network cells. The height of the pattern over a given cell denotes the importance of its feature at that time. As this is happening, the input pattern uses parallel pathways to generate excitatory signals to the orienting system *A* with a gain ρ that is called the *vigilance* parameter.

Activity pattern *X* generates inhibitory signals to the orienting system *A* while it also activates bottom-up excitatory pathways that transmit an input pattern *S* to the category level *F*_2_. A dynamic balance within *A* emerges between excitatory inputs from *I* and inhibitory inputs from *S* that keeps *S* quiet while level *F*_2_ is getting activated.

The bottom-up signals *S* in pathways from the feature level *F*_1_ to the category level *F*_2_ are multiplied by learned adaptive weights at the ends of these pathways to form the input pattern *T* to *F*_2_. The inputs *T* are contrast-enhanced and normalized within *F*_2_ by recurrent lateral inhibitory signals that obey the membrane equations of neurophysiology, also called shunting interactions. This competition selects a small number of cells within *F*_2_ that receive the largest inputs. The chosen cells represent the category Y that codes the feature pattern at *F*_1_. In Figure 3A, a winner-take-all category is shown.

As depicted in Figure 3B, the activated category *Y* then generates top-down signals *U*. These signals are also multiplied by adaptive weights to form a prototype, or *critical feature pattern, V* which encodes the expectation that is learned by the active *F*_2_ category of what feature pattern to expect at *F*_1_. This top-down expectation input *V* is added at *F*_1_ cells using the ART Matching Rule (Figure 2). The ART Matching Rule is realized by a top-down, modulatory on-center, off-surround network that focuses attention upon its critical feature pattern. This matching process, repeated over a series of learning trials, determines what critical features will be learned and chosen in response to each category in the network.

Also shown in Figure 3B is what happens if *V* mismatches *I* at some cells in *F*_1_. Then a subset of the features in *X*—denoted by the STM activity pattern X^*^ (the pattern of gray features)—is selected at cells where the bottom-up and top-down input patterns match well enough. In this way, X^*^ is active at *I* features that are confirmed by *V*, at the same time that mismatched features (white region of the feature pattern) are inhibited. When *X* changes to X^*^, the total inhibitory signal from *F*_1_ to *A* decreases, thereby setting the stage for the hypothesis testing cycle.

In particular, as in Figure 3C, if inhibition decreases sufficiently, the orienting system *A* generates a non-specific arousal burst (denoted by Reset) to *F*_2_. This event mechanizes the intuition that “novel events are arousing.” The vigilance parameter ρ, which is computed in *A*, determines how bad a match will be tolerated before non-specific arousal is triggered. Each arousal burst initiates hypothesis testing and a memory search for a better-matching category. This happens as follows:

First, arousal resets *F*_2_ by inhibiting the active category cells *Y* (*Y* is crossed out in Figure 3C). After *Y* is inhibited, then, as denoted by the x's over the top-down pathways in Figure 3C, the top-down expectation *V* shuts off too, thereby removing inhibition from all feature cells in *F*_1_. As shown in Figure 3D, pattern *X* is disinhibited and thereby reinstated at *F*_1_. Category *Y* stays inhibited as *X* activates a different category Y^*^ at *F*_2_. This memory search cycle continues until a better matching, or novel, category is selected.

As learning dynamically stabilizes, inputs *I* directly activate their globally best-matching categories directly through the adaptive filter, without activating the orienting system. Grossberg (2017b) summarizes psychological and neurobiological data that support each of these processing stages.

### 2.7. Feature-Category Resonances, Conscious Recognition, and Explainable Attended Features

**Figure 4:**
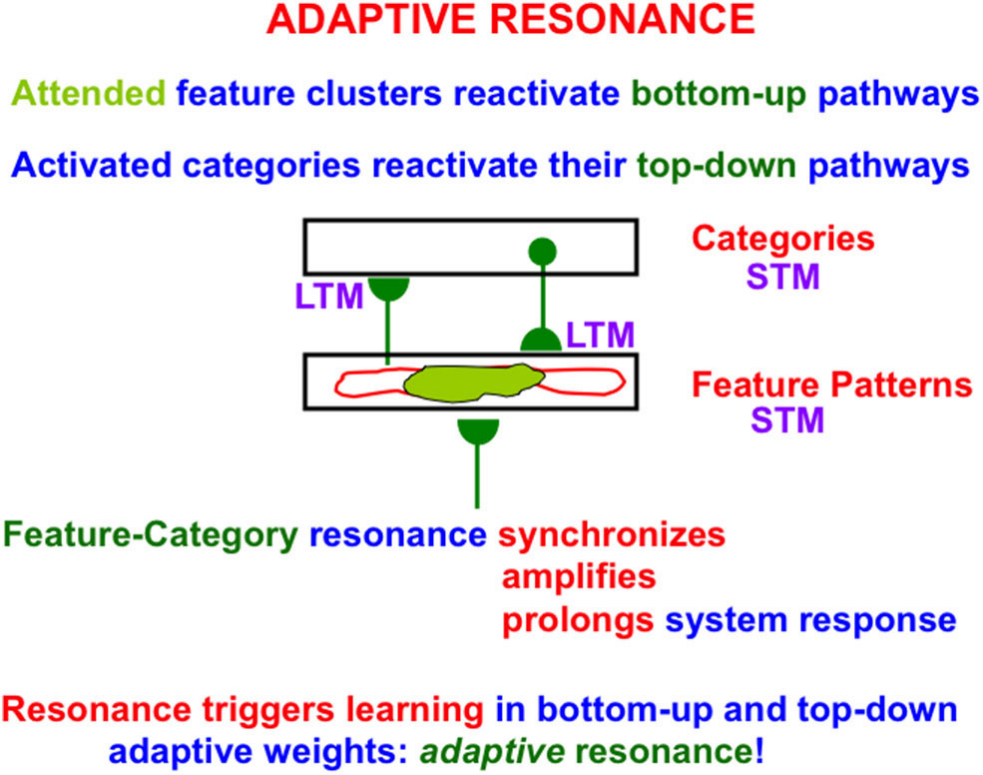
When a good enough match occurs between a bottom-up input pattern and top-down expectation, a feature-category resonance is triggered the synchronizes, amplifies, and prolongs the STM activities of the cells that participate in the resonance, while also selecting an attentional focus and triggering learning in the LTM traces in the active bottom-up adaptive filter and top-down expectation pathways to encode the resonating attended data [adapted with permission from Grossberg (2017b)].

When search ends, a *feature-category resonance* develops between the chosen activities, or short-term memory (STM) traces, of the active critical feature pattern and recognition category (Figure 4). Such a feature-category resonance synchronizes, amplifies, and prolongs the activities within the critical feature pattern, and supports conscious recognition of the chosen category.

This resonance also triggers learning in the adaptive weights, or long-term memory (LTM) traces, within the active bottom-up and top-down pathways; hence the name *adaptive* resonance. The attended critical feature pattern at *F*_1_ hereby learns to control what features are represented by the currently active category *Y* in Figure 3. Inspecting an active critical feature pattern can “explain” what its category has learned, and what features activation of this category will prime in the future using top-down signals. Looking at the critical feature patterns also explains what features will control intermodal predictions that are formed via supervised learning and are read-out by this category (see Section 2.10 and Figure 8 below).

### 2.8. Catastrophic Forgetting Without the Top-Down ART Matching Rule

Before turning to intermodal predictions, I will provide examples of how learned top-down expectations prevent catastrophic forgetting, and how vigilance controls how specific or general the categories that are learned become. The intuitive idea about how top-down expectations in ART avoid catastrophic forgetting is that ART learns critical feature patterns of LTM weights in both its bottom-up adaptive filters and its top-down expectations. ART can hereby focus attention upon predictively relevant data (Figure 4) while inhibiting outliers that could otherwise have caused catastrophic forgetting.

**Figure 5:**
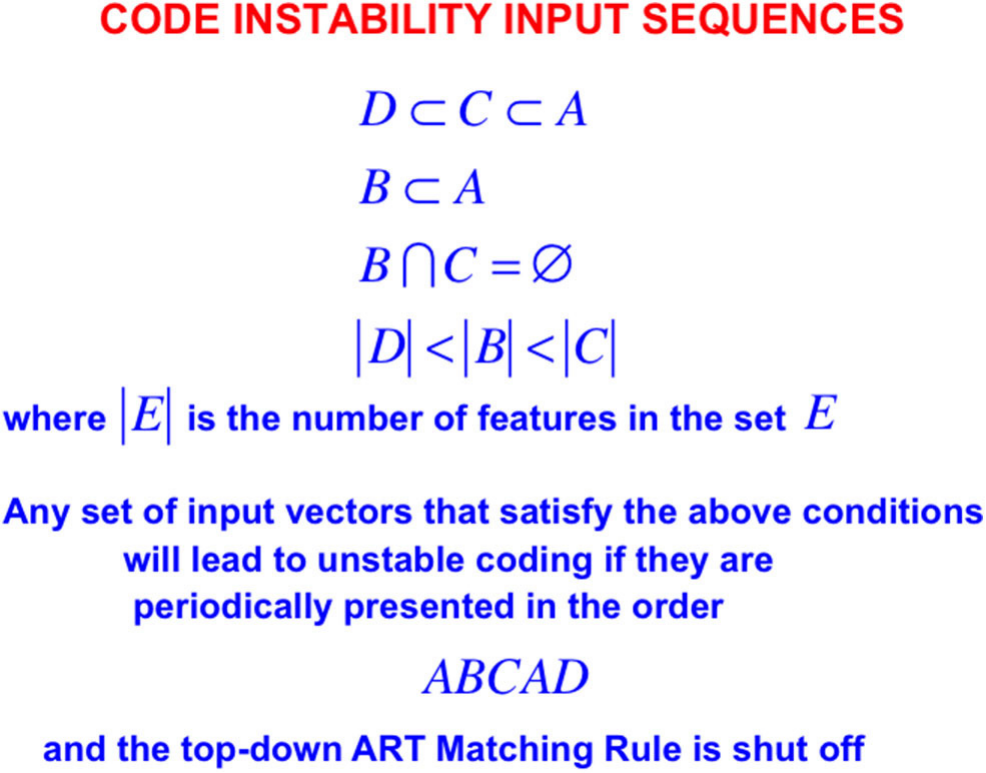
When the ART Matching Rule is eliminated by deleting an ART circuit's top-down expectations from the ART 1 model, the resulting competitive learning network experiences catastrophic forgetting even if it tries to learn any of arbitrarily many lists consisting of just four input vectors A, B, C, and D when they are presented repeatedly in the order ABCAD, assuming that the input vectors satisfy the constraints shown in the figure [adapted with permission from Carpenter and Grossberg (1987)].

**Figure 6:**
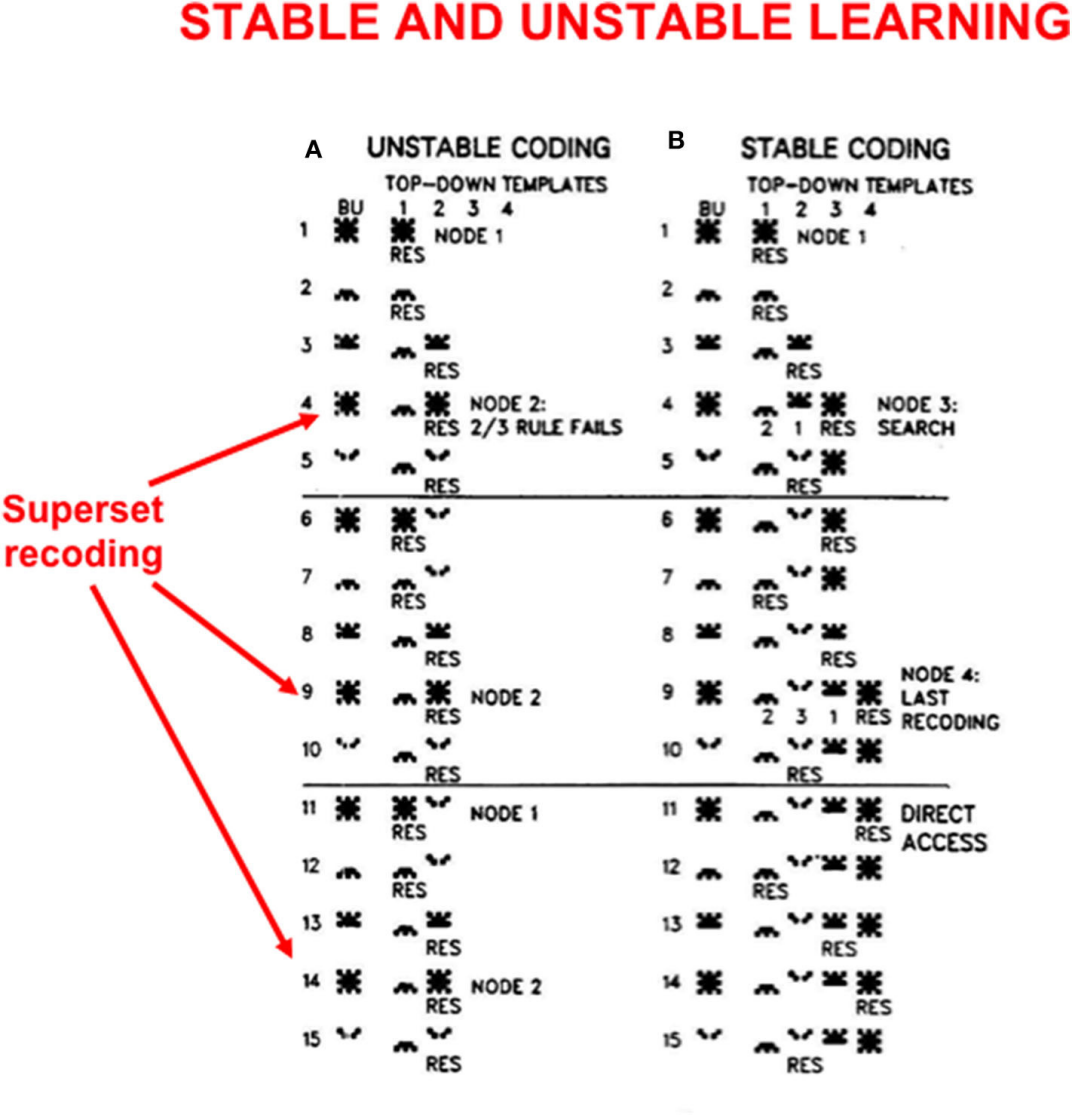
These computer simulations illustrate how **(A)** unstable learning and **(B)** stable learning occur in response to a particular sequence of input vectors A, B, C, D when they are presented repeatedly in the order ABCAD to an ART 1 model. Unstable learning with catastrophic forgetting of the category that codes vector A occurs when no top-down expectations exist, as illustrated by its periodic recoding by categories 1 and 2 on each learning trial. See the text for details [adapted with permission from Carpenter and Grossberg (1987)].

In order to see more clearly how top-down expectations prevent catastrophic forgetting, a set of simulations that were carried out using the ART 1 model by Carpenter and Grossberg (1987) will be summarized. Carpenter and Grossberg (1987) illustrated how easy it is for catastrophic forgetting to occur by describing a class of infinitely many sequences of input patterns whose learning exhibits catastrophic forgetting if top-down expectations that obey the ART Matching Rule are eliminated. In fact, sequences of just four input patterns, suitably ordered, lead to catastrophic forgetting in the absence of top-down expectations. Figure 5 summarizes the mathematical rules that generate such sequences. Figure 6A summarizes a computer simulation that demonstrates catastrophic forgetting when top-down matching is eliminated. Figure 6B illustrates how stable learning is achieved when top-down matching is restored. As Figure 6A illustrates, unstable coding can occur if a learned subset prototype gets recoded as a superset prototype when a superset input pattern is categorized by that category.

This kind of recoding happens every time any sequence of four input patterns that is constrained by the rules in Figure 5 is presented in the order *ABCAD*. Note that pattern *A* is a superset of each of the other patterns *B, C*, and *D* in the sequence. Pattern *A* is presented as the first and the fourth input in the sequence *ABCAD*. When it is presented as the first input, it is categorized by category node 1 in Figure 6A, but when it is presented as the fourth input, it is categorized by category node 2. This oscillatory recoding occurs on each presentation of the sequence, so *A* is catastrophically recoded on every learning trial. The simulation in Figure 6B shows that restoring the ART Matching Rule prevents this kind of “superset recoding.”

### 2.9. Vigilance Regulates Learning of Concrete and General Category Prototypes

**Figure 7:**
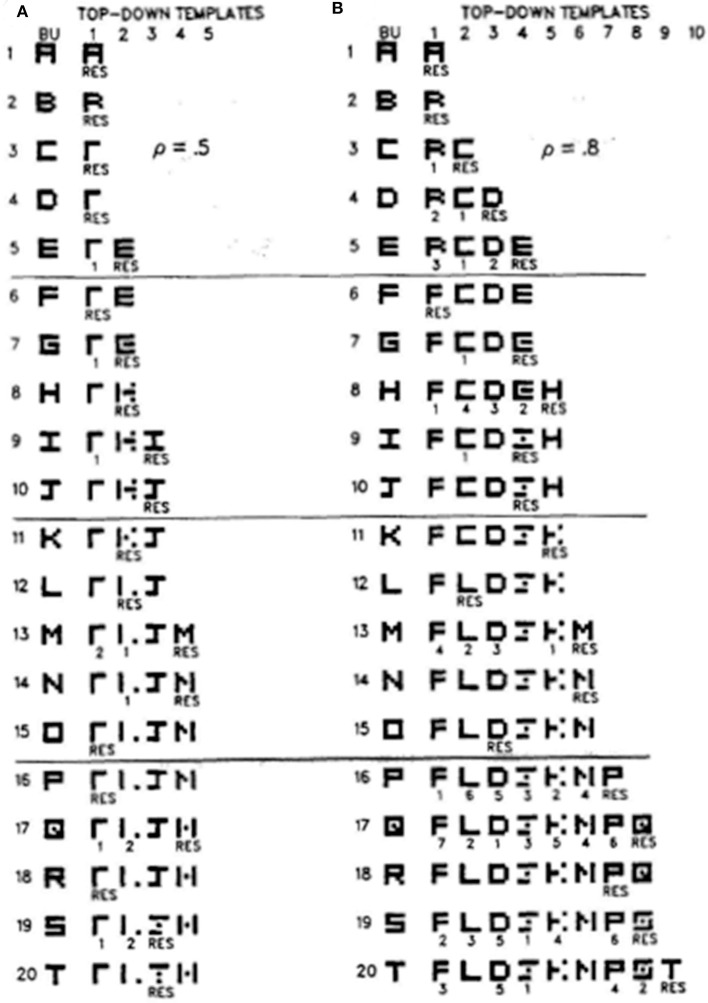
These computer simulations show how the alphabet A, B, C, … is learned by the ART 1 when vigilance is chosen to equal **(A)** 0.5, or **(B)** 0.8. Note that more categories are learned in **(B)** and that their learned prototypes more closely represent the letters that they categorize. Thus, higher vigilance leads to the learning of more concrete categories. See the text for details [reprinted with permission from Carpenter and Grossberg (1987)].

Figure 7 illustrates how different vigilance levels in a single ART model can lead to learning of both concrete and general category prototypes, and thus categories that can code a small number of very similar exemplars (concrete) or a large number of only vaguely similar exemplars (general). This figure summarizes the results of computer simulations in Carpenter and Grossberg (1987) showing how the ART 1 model can learn to classify the letters of the alphabet. During alphabet learning in real life, the raw letters would not be directly input into the brain's recognition categories in the inferotemporal cortex. They would first be preprocessed by visual cortex in the manner summarized in Grossberg (2020). How vigilance control works is, however, vividly shown by inputting letters directly to the ART classifier.

If vigilance is set to its maximum value of 1, then no variability in a letter is tolerated, and every letter is classified into its own category. This is the limit of exemplar prototypes. Figure 7A shows how the letters are classified if vigilance is set at a smaller value 0.5. Figure 7B shows the same thing if vigilance is set at a larger value 0.8. In both cases, the network's learning rate is chosen to be high.

Going down the column in Figure 7A shows how the network learns in response to the first 20 letters of the alphabet when vigilance equals 0.5. Each row describes what categories and prototypes are learned through time. Black pixels represent prototype values equal to 1 at the corresponding positions. White pixels represent prototype values equal to 0 at their positions. Scanning down the learning trials 1, 2,…20 shows that each prototype becomes more abstract as learning goes on. By the time letter T has been learned, only four categories have been learned with which to classify all 20 letters. The symbol RES, for resonance, under a prototype on each learning trial shows which category classifies the letter that was presented on that trial. In particular, category 1 classifies letters A, B, C, and D, among others, when they are presented, whereas category 2 classifies letters E, G, and H, among others, when they are presented.

Figure 7B shows that, when the vigilance is increased to 0.8, nine categories are learned in response to the first 20 letters, instead of four. Letter C is no longer lumped into category 1 with A and B. Rather, it is classified by a new category 2 because it cannot satisfy vigilance when it is matched against the prototype of category 1. Together, Figures 7A,B show that, just by changing the sensitivity of the network to attentive matches and mismatches, it can either learn more abstract or more concrete prototypes with which to categorize the world.

Figure 7 also provides examples of how memory search works. During search, arousal bursts from the orienting system interact with the attentional system to rapidly reset mismatched categories, as in Figure 3C, and to thereby allow selection of better *F*_2_ representations with which to categorize novel inputs at *F*_1_, as in Figure 3D. Search may end with a familiar category if its prototype is similar enough to the input exemplar to satisfy the resonance criterion. This prototype may then be refined by attentional focusing to incorporate the new information that is embodied in the exemplar. For example, in Figure 7A, the prototype of category 1 is refined when B and C are classified by it. Likewise, the prototype of category 2 is refined when G, H, and K are classified by it. If, however, the input is too different from any previously learned prototype, then an uncommitted population of *F*_2_ nodes is selected and learning of a new category is initiated. This is illustrated in Figure 7A when E is classified by category 2 and when I is classified by category 3. Search hereby uses vigilance control to determine how much a category prototype can change and, within these limits, protects previously learned categories from experiencing catastrophic forgetting.

The simulations in Figure 7 were carried out using unsupervised learning. Variants of ARTMAP models can learn using arbitrary combinations of unsupervised and supervised learning trials. Fuzzy ARTMAP illustrates how this happens in how this happens in Section 2.10.

### 2.10. How Learning Starts: Small Initial Bottom-Up Weights and Large Top-Down Weights

In a self-organizing system like ART that can learn in an unsupervised way, an important issue is: How does learning get started? This issue does not arise in systems, such as back propagation and Deep Learning, where the correct answer is provided on every supervised learning trial to back-propagate teaching signals that drive weights slowly toward target values (Figure 1). Here is how both bottom-up and top-down adaptive weights work during ART unsupervised learning:

Bottom-up signals within ART adaptive filters from feature level *F*_1_ (Figure 3A) are typically gated, before learning occurs, by small and randomly chosen adaptive weights before they activate category level *F*_2_. When *F*_2_ receives these gated signals from *F*_1_, recurrent on-center off-surround signals within *F*_2_ choose a small subset of cells that receive the largest inputs. This recurrent network also contrast-enhances the activities of the winning cells, while normalizing the total STM-stored activity across the network. The small bottom-up inputs can hereby generate large enough activities in the winning *F*_2_ cells for them to drive efficient learning in their abutting synapses.

Initial top-down learning faces a different problem: How does a top-down expectation of a newly discovered recognition category learn how to match the feature pattern that activates it, given that the category has no idea what feature pattern this is? This can happen because all of its top-down adaptive weights initially have large values, and can thereby match *any* feature pattern. As learning proceeds, these broadly distributed adaptive weights are *pruned* to incrementally select the appropriate attended critical feature pattern for that category.

### 2.11. Fuzzy ARTMAP: A Self-Organizing Production and Rule Discovery System

**Figure 8:**
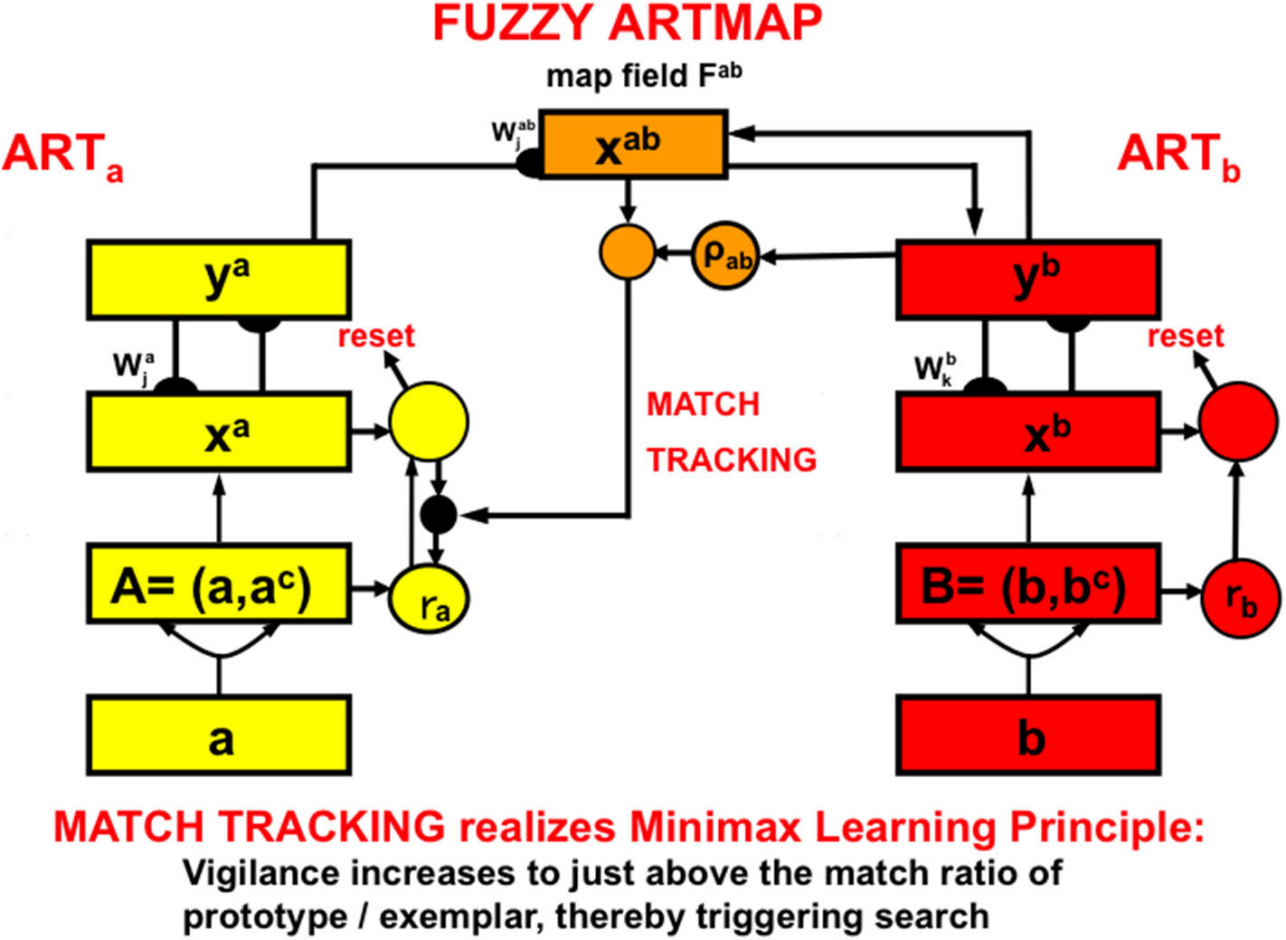
The fuzzy ARTMAP architecture can learn recognition categories in both ART_a_ and ART_b_ by unsupervised learning, as well as an associative map via the map field from ART_a_ to ART_b_ by supervised learning. See the text for details [adapted with permission from Carpenter et al. (1992)].

The fuzzy ARTMAP model (Figure 8; Carpenter et al., 1992; Carpenter and Markuzon, 1998) is an ART model that incorporates some of the operations of fuzzy logic. Fuzzy ARTMAP enables maps to be learned from a pair of ART category learning networks, ARTa and ARTb, that can each be trained using unsupervised learning. The choices of ARTa and ARTb include a huge variety of possibilities.

For example, the categories learned by ARTa can represent visually processed objects, whereas the categories learned by ARTb can represent the auditorily processed names of these objects. An associative mapping from ARTa to ARTb, mediated by a map field F^ab^ (Figure 8), can then learn to predict the correct name of each object. This kind of intermodal map learning illustrated how supervised learning can occur in ARTMAP.

In the present example, after map learning occurs, inputting a picture of an object into ARTa can predict its name via ARTb because each of ARTa and ARTb learns via bottom-up adaptive filter pathways *and* top-down expectation pathways. If a sufficiently similar picture has been learned in the past, its presentation to level x^a^ in ARTa can activate a visual recognition category in y^a^. This category can then use the learned association from ARTa to ARTb to activate a category of the object's name in y^b^. Then a learned top-down expectation from the auditory name category can activate a representation in x^b^ of the auditory features that characterize the name.

In a very different application, the categories learned by ARTa can include disease symptoms, treatments for them, and medical test results, whereas the categories learned by ARTb can represent the predicted length of stay in the hospital in response to different combinations of these factors. Being able to predict this kind of outcome in advance can be invaluable in guiding hospital planning.

A hospital's willingness to trust such predictions is bolstered by the fact that the adaptive weights of fuzzy ARTMAP can, at any stage of learning, be translated into fuzzy IF-THEN rules that allow practitioners to understand the nature of the knowledge that the model has learned, as well as the amount of variability in the data that each of the learned rules can tolerate. As will be explained more completely in completely in Section 2.13, these IF-THEN rules “explain” the knowledge upon which the predictions have been made. The learned categories themselves play the role of symbols that compress this rule-based knowledge and can be used to read out predictions based upon them. Fuzzy ARTMAP is thus a self-organizing production and *rule discovery* system, as well as a neural network that can learn symbols with which to predict changing environments.

Neither back propagation nor Deep Learning enjoys any of these properties. The next property, no less important, also has no computational counterpart in either of these algorithms.

### 2.12. Minimax Learning via Match Tracking: Maximize Generalization and Minimize Error

In fuzzy ARTMAP, as with all ART models, vigilance is initially set to be as low as possible before learning begins. A low initial setting of vigilance enables the learning of large and general categories. General categories conserve memory resources, but may do so at the cost of allowing too many predictive errors. ART proposes how to use vigilance control to conjointly maximize category generality while minimizing predictive errors by a process called *match tracking* that realizes a *minimax learning rule*.

**Figure 9:**
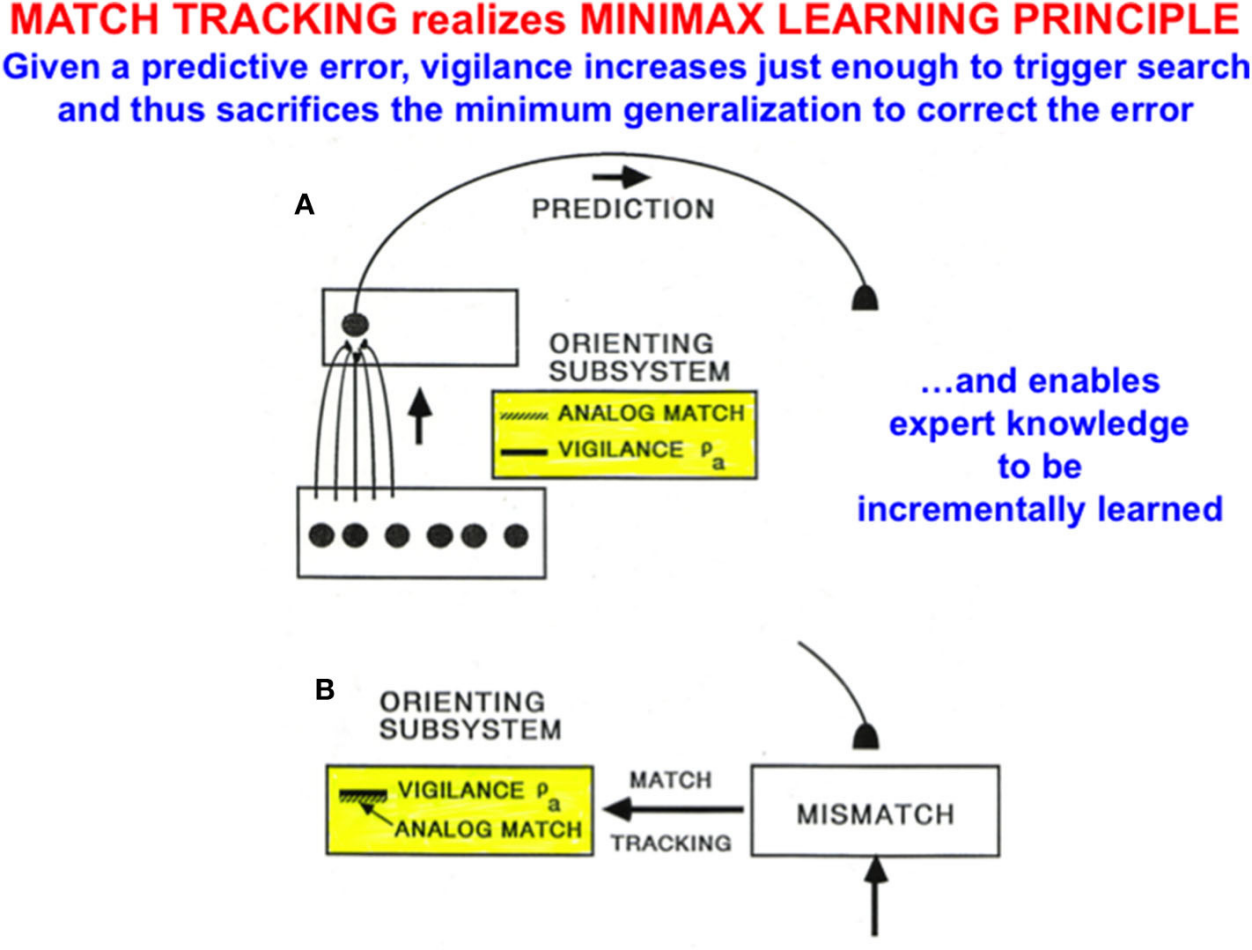
**(A)** A prediction from ART_a_ to ART_b_ can be made if the analog match between bottom-up and top-down patterns exceeds the current vigilance value. **(B)** If a mismatch occurs between the prediction at ART_b_ and the correct output pattern, then a match tracking signal can increase vigilance just enough to drive hypothesis testing and memory search for a better-matching category at ART_a_. Matching tracking hereby sacrifices the minimum amount of generalization necessary to correct the predictive error [adapted with permission from Carpenter and Grossberg (1992)].

Match tracking works as follows (Figure 9): Suppose that an incorrect prediction is made from ARTb in response to an input vector to ARTa on a supervised learning trial. In order for any prediction to have been made, the *analog match* in ARTa between, as described in Figure 8, the bottom-up input A = (a, a^c^) to x^a^ and the top-down expectation from y^a^ to x^a^ must exceed the vigilance parameter ρ_*a*_ at that time, as in Figure 9A. The mismatch in ARTb (see Figure 8) between the actual output vector that is read out by y^b^, and desired output vector B = (b, b^c^) from the environment, then triggers a *match tracking* signal from ARTb, via the map field F^ab^, to ARTa. The vectors A = (a, a^c^) and B = (b, b^c^) are normalized by complement coding, where a^c^ = 1-a and b^c^ = 1-b.

The match tracking signal derives its name from the fact that it increases the vigilance parameter until it just exceeds the analog match value (Figure 9B). In other words, vigilance “tracks” the analog match value in ARTa. When vigilance exceeds the analog match value, it can activate the orienting system *A*, for reasons that will immediately be explained. A new bout of hypothesis testing and memory search is then triggered to discover a better category with which to predict the mismatched outcome.

Since each increase in vigilance causes a reduction in the generality of learned categories, increasing vigilance via match tracking causes the minimum reduction in category generality that can correct the predictive error. Category generality and predictive error hereby realize *minimax learning*: Maximizing category generality while conjointly minimizing predictive error.

### 2.13. How Increasing Vigilance Can Trigger Memory Search

A simple mathematical inequality explains both how vigilance works, and why it is computed in the orienting system *A*. Since back propagation and Deep Learning do not have an orienting system, they cannot compute a parameter like vigilance in an elegant way.

Vigilance is computed in the orienting system (Figures 3B–D) because it is here that bottom-up excitation from all the active inputs in an input pattern *I* are compared with inhibition from all the active features in a distributed feature representation across *F*_1_. In particular, the vigilance parameter ρ multiplies the bottom-up inputs *I* to the orienting system *A*; thus, ρ is the *gain*, or sensitivity, of the excitatory signals that the inputs *I* deliver to *A*. The total strength ρ|*I*| of the active excitatory input to *A* is inhibited by the total strength |X^*^| of the current activity at *F*_1_.

Memory search is prevented, and resonance allowed to develop, if the net input ρ|*I*| − |X^*^| to the orienting system from the attentional system is ≤ 0. Then the total output |X^*^| from active cells in the attentional focus X^*^ *inhibits* the orienting system *A* (note the minus sign) in ρ|*I*| − |X^*^| more than the total input ρ|*I*| at that time *excites* it.

If |X^*^| is so small that ρ|*I*| − |X^*^| becomes positive, then the orienting system *A* is activated, as in Figure 3C. The inequality ρ|*I*| − |X^*^| > 0 can be rewritten as ρ > |X^*^||*I*|^−1^ to show that the orienting system is activated whenever ρ is larger than the ratio of the number of active matched features in X^*^ to the total number of features in *I*. In other words, the vigilance parameter controls how bad a match can be before search for a new category is initiated. If the vigilance parameter is low, then many exemplars can all influence the learning of a shared prototype, by chipping away at the features that are not shared with all the exemplars. If the vigilance parameter is high, then even a small difference between a new exemplar and a known prototype (e.g., letter *F* vs. *E*) can drive the search for a new category with which to represent *F*.

Either a larger value of the vigilance ρ, or a smaller match ratio |X^*^||*I*|^−1^ makes it harder to achieve resonance. This is true because, when ρ is larger, it is easier to make ρ|*I*| − |X^*^| positive, thereby activating the orienting system and leading to memory search. A large vigilance hereby makes the network more intolerant of differences between the input and the learned prototype. Alternatively, for fixed vigilance, if the input is chosen to be increasingly different from the learned prototype, then X^*^ becomes smaller and the match ratio ρ|*I*| − |X^*^| becomes larger until ρ|*I*| − |X^*^| becomes positive, and a memory search is triggered by a burst of arousal.

### 2.14. Learned Fuzzy IF-Then Rules in Fuzzy ARTMAP Explain Its Categories

The algebraic equations that define fuzzy ARTMAP will not be reviewed here. Instead, I will just note that each adaptive weight vector has a geometric interpretation as a rectangle (or hyper-rectangle in higher dimensions) whose corners in each dimension represent the extreme values of the input feature which that dimension represents, and who size represents the degree of fuzziness that input vectors which code that category can have and still remain within it.

If a new input vector falls outside the rectangle on a supervised learning trial, but does not trigger category reset and hypothesis testing for a new category, then the rectangle is expanded to become the smallest rectangle that includes both the previous rectangle and the newly learned vector, unless this new rectangle becomes too large. The maximal size of such learned rectangles has an upper bound that increases as vigilance decreases, so that more general categories can be learned at lower vigilance.

Inspection of such hyper-rectangles provides immediate insight into both the feature vectors that control category learning and predictions, and how much feature variability is tolerated before category reset and hypothesis testing for another category will be triggered.

## 3. Explainable Visual and Auditory Percepts

### 3.1. Biological Vision: Completed Boundaries Gate Filling-in Within Depth-Selective Surfaces

Perhaps the most “explainable” representations in biological neural models are those that represent perceptual experiences, notably visual and auditory percepts. The functional units of visual perception are boundaries and surfaces, whose formation and properties have been modeled to explain and predict a wide variety of psychological and neurobiological data about how humans and other primates (see Grossberg, 1987a,b, 1994, 1997; Grossberg and Raizada, 2000; Kelly and Grossberg, 2000; Grossberg et al., 2002, 2007; Grossberg and Howe, 2003; Grossberg and Swaminathan, 2004; Cao and Grossberg, 2005, 2012; Grossberg and Yazdanbakhsh, 2005; Grossberg and Hong, 2006). Visual boundaries are formed when perceptual groupings are completed in response to the spatial distribution of edges, textures, and shading in images and scenes. Visual surfaces are formed when brightnesses and colors fill-in within these completed boundaries after they have discounted the distorting effects of spatially variable illumination levels.

**Figure 10:**
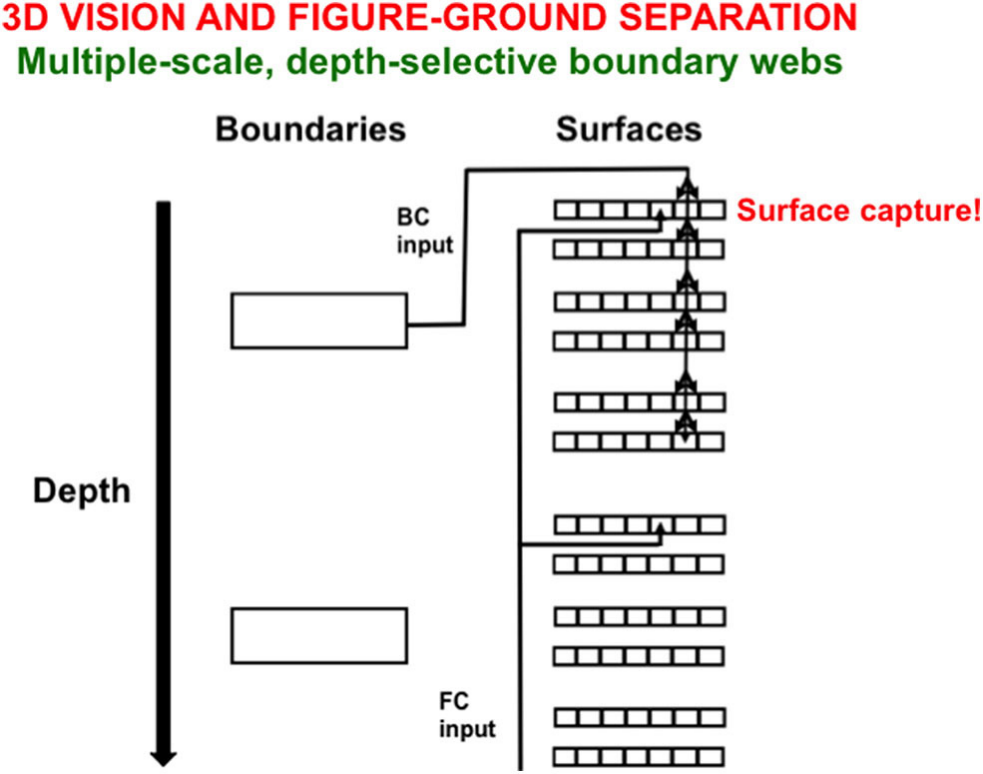
Spatially abutting and collinear boundary contour (BC) and feature contour (FC) signals in a Filling-In-DOmain, or FIDO, can trigger depth-selective filling-in of the color carried by the FC signal in that FIDO. See the text for details [adapted with permission from Grossberg and Zajac (2017)].

Figure 10 illustrates one step in the process whereby boundaries capture surfaces at different relative depths from an observer. Here, depth-selective boundary representations are completed at different depths. Each boundary representation generates topographic boundary inputs, called Boundary Contour (BC) signals, to all the multiple color-selective surface representations (red, green; blue, yellow; black, white) at its depth. Color signals from which the illuminant has been discounted are also topographically input to their color-selective surface filling-in domains across all the depths. These color signals are called Feature Contour (FC) signals because they represent the “features” that may eventually become consciously visible.

A BC input can capture the color represented by an FC input if both contours are spatially contiguous and orientationally aligned within a particular depth-selective Filling-In Domain, or FIDO. When this happens, the color will selectively fill in the boundaries of that FIDO. It is in this way that FC signals that are broadcast to all surface depths capture colored surface percepts within some depth-selective FIDOs but not others.

Various boundary completion and surface-to-boundary feedback processes, among others, are needed to complete depth-selective filled-in surface representations. The articles cited above explain in detail how this happens. How all these boundary and surface interactions occur and explain many interdisciplinary data about vision is the central explanatory goal of the Form-And-Color-And-DEpth, or FACADE, theory of 3D vision and figure-ground perception. FACADE theory derives its name from the prediction, which is supported by a great deal of perceptual and neurobiological evidence, that surface representations multiplex properties of form, color, and depth in prestriate visual cortical area V4. The qualia that are supported by these surface representations are, in principle, explainable by measurements using parallel arrays of microelectrodes in the relevant cortical areas.

### 3.2. Surface-Shroud Resonances for Conscious Seeing and Reaching

These surface representations are predicted to become consciously seen when they form part of *surface-shroud resonances* that occur between cortical areas V4 and the posterior parietal cortex, or PPC. Grossberg (2017b) reviews model processes along with psychological and neurobiological data that support this prediction, including clinical data about how parietal lesions—by disrupting the formation of surface-shroud resonances—lead to visual neglect (Driver and Mattingley, 1998; Mesulam, 1999; Bellmann et al., 2001; Marshall, 2001), visual agnosia (Goodale et al., 1991; Goodale and Milner, 1992), and impairments of sustained visual attention (Robertson et al., 1997; Rueckert and Grafman, 1998). Problems with motor planning, notably reaching deficits, also occur as a result of parietal lesions (Heilman et al., 1985; Mattingley et al., 1998), in keeping with the hypothesis that “we see in order to look and reach.” Thus, the dual roles of parietal cortex—focusing and maintaining spatial attention, and directing motor intention—are both disrupted by parietal lesions.

The resonating surface representation in V4 during a surface-shroud resonance is explainable in principle, especially when it is correlated with the visual qualia that it represents. So too is the attentional shroud in PPC that configures its spatial extent to cover, or shroud, the surface representation with which it is resonating.

### 3.3. Stream-Shroud Resonances for Conscious Hearing and Auditory Communication

Grossberg (2017b) also summarizes theoretical, psychological, and neurobiological evidence for the assumption that *stream-shroud resonances* arise in the auditory system, where they support conscious hearing and auditory communication, including speech and language. Stream-shroud resonances are predicted to be homologous to the surface-shroud resonances in the visual system, although visual surfaces represent physical space, whereas auditory streams represent frequency space. Parietal lesions that undermine stream-shroud resonances lead to clinical data that are similar to those in the visual system, including neglect, agnosia, attentional problems, and problems of auditory communication.

Auditory streams separate different acoustic sources in the environment so that they can be tracked and learned about, a process that is often called *auditory scene analysis* (Bregman, 1990). Perhaps the most famous example of streaming occurs in solving the classical *cocktail party problem* (Cherry, 1953), which asks how listeners can hear an attended speaker even if the frequencies in her voice are similar to the frequencies of nearby speakers or are occluded by intermittent background noise. Grossberg et al. (2004) introduced the ARTSTREAM neural model to explain design principles and mechanisms whereby our brains track acoustic sources whose frequencies overlap and may be occluded by intermittent noise. Auditory streams are explainable because our brains generate spatially distinct frequency representations over which auditory streams can be followed through time. The parietal shrouds in stream-shroud resonances cover auditory streams just as the shrouds in surface-shroud resonances cover visual surfaces, and thus are also explainable.

## 4. Explainable Emotions During Cognitive-Emotional Interactions

### 4.1. Where Cognition and Emotion Meet: Conditioning and Cognitive-Emotional Resonances

In addition to explainable representations of perception and cognition, explainable representations of emotion and cognitive-emotional interactions have also been modeled. These models explain how events in the world learn to activate emotional reactions, how emotions can influence the events to which attention is paid, and how emotionally salient events can learn to trigger responses aimed at acquiring valued goals. This kind of cognitive-emotional learning is often accomplished by either classical conditioning, also called Pavlovian conditioning (Pavlov, 1927), or operant conditioning, also called instrumental or Skinnerian conditioning (Skinner, 1938).

The current review will discuss only classical conditioning models of cognitive-emotional learning, although the same models also perform appropriately during operant conditioning. During classical conditioning, a neutral sensory event, such as a tone or a light, called the *conditioned stimulus* (CS), is associated with an emotionally-charged event, such as the presentation of food or shock, called the *unconditioned stimulus* (US). The US typically elicits a reflexive response, such as eating or withdrawal, called the *unconditioned response* (UR). Pairing a CS a sufficient number of times at an appropriate time interval before a US can elicit a learned response, called the *conditioned response* (CR) that is similar to the UR.

**Figure 11:**
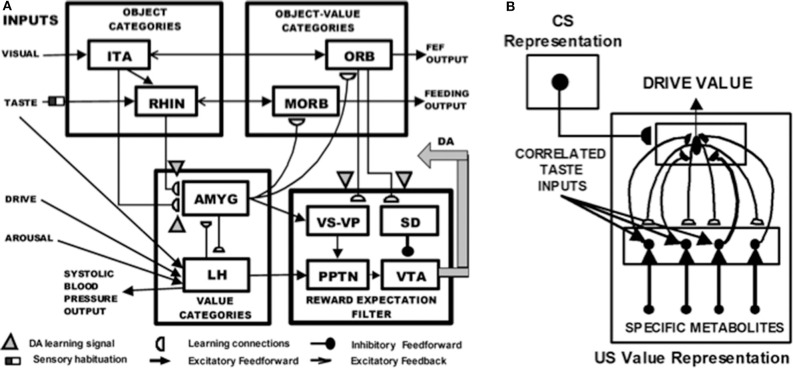
**(A)** Object categories are activated by visual or gustatory inputs in anterior inferotemporal (ITA) and rhinal (RHIN) cortices, respectively. Value categories represent the value of anticipated outcomes on the basis of current hunger and satiety inputs in amygdala (AMYG) and lateral hypothalamus (LH). Object-value categories occur in the lateral orbitofrontal (ORB) cortex, for visual stimuli, and the medial orbitofrontal (MORB) cortex, for gustatory stimuli. They use the learned value of perceptual stimuli to choose the most valued stimulus in the current context. The reward expectation filter in the basal ganglia detects the omission or delivery of rewards using a circuit that spans ventral striatum (VS), ventral pallidum (VP), striosomal delay (SD) cells in the ventral striatum, the pedunculopontine nucleus (PPTN), and midbrain dopaminergic neurons of the substantia nigra pars compacta/ventral tegmental area (SNc/VTA). **(B)** Reciprocal excitatory signals from hypothalamic drive-taste cells to amygdala value category cells can drive the learning of a value category that selectively fires in response to a particular hypothalamic homeostatic activity pattern. See the text for details [adapted with permission from Dranias et al. (2008)].

Figure 11A depicts the macrocircuit of the MOTIVATOR neural model, which explains many psychological and neurobiological data in this area (Grossberg, 1975, 1978, 1984, 2018; Brown et al., 1999, 2004; Dranias et al., 2008; Grossberg et al., 2008; Silver et al., 2011; Grossberg and Kishnan, 2018). The MOTIVATOR model describes how four types of brain processes interact during conditioning and learned performance: *Object categories* in the anterior inferotemporal (ITA) cortex and the rhinal (RHIN) cortex, *value categories* in the amygdala (AMYG) and lateral hypothalamus (LH), *object-value categories* in the lateral (ORB) and medial orbitofrontal (MORB) cortices, and a *reward expectation filter* in the basal ganglia, notably in the substantia nigra pars compacta (SNc) and the ventral tegmental area (VTA). Only the VTA is shown in Figure 11A.

A visual CS activates an object category in the ITA, whereas a US activates a value category in the AMYG. During classical conditioning in response to CS-US pairings, *conditioned reinforcer learning* occurs in the ITA-to-AMYG pathway, while *incentive motivational learning* occurs in the AMYG-to-ORB pathway. After a CS becomes a conditioned reinforcer, it can cause many of the internal emotional reactions and responses at the AMYG that its US could, by activating the same internal drive representation there. After the CS activates a drive representation in the AMYG, this drive representation triggers incentive motivational priming signals via the AMYG-to-ORB pathway to ORB cells that are compatible with that drive. In all, when the CS is activated, it can send signals directly to its corresponding ORB representation, as well as indirectly via its learned ITA-to-AMYG-to-ORB pathway. These converging signals enable the recipient ORB cells to fire and trigger a CR. All of these adaptive connections end in hemidiscs, which house the network's LTM traces.

The representations in ITA, AMYG, and ORB are explainable because presentation of a visual CS will be highly correlated with selective activation of its ITA object category, presentation of a reinforcer US will be highly correlated with selective activation of its AMYG value category, and the simultaneous activation of both ITA and AMYG will be highly correlated with selective activation of the ORB representation that responds to this particular object-drive combination.

The basal ganglia carries out functions that are computationally complementary to those of the amygdala, thereby enabling the system as a whole to cope with both expected and unexpected events: In particular, as explained above, the AMYG generates incentive motivational signals to the ORB object-value categories to trigger previously learned actions in *expected* environmental contexts. In contrast, the basal ganglia generates Now Print signals that drive new learning in response to *unexpected* rewards. These Now Print signals release dopamine (DA) signals from SNc and VTA to multiple brain regions, where they modulate learning of new associations there.

When the feedback loop between object, value, and object-value categories is closed by excitatory signals, then this circuit goes into a *cognitive-emotional resonance* that supports conscious recognition of an emotion and the object that has triggered it.

### 4.2. Where Cognition and Emotion Meet: Conditioning and Drive-Value Resonances

Figure 11B shows how reciprocal adaptive connections between LH and AMYG enable AMYG cells to become learned value categories that are associated with particular emotional qualia. The AMYG interacts reciprocally with taste-drive cells in the LH at which taste and metabolite inputs converge. Bottom-up signals from activity patterns across LH taste-drive cells activate competing value categories in the AMYG. A winning AMYG value category learns to respond selectively to specific combinations of taste-drive activity patterns and sends adaptive top-down expectation signals back to the taste-drive cells that activated it. The activity pattern across LH taste-drive cells provides an explainable representation of the homeostatic factors that subserve a particular emotion.

When the reciprocal excitatory feedback pathways between the hypothalamic taste-drive cells and the amygdala value category are activate, they generate a *drive-value resonance* that supports the conscious emotion which corresponds to that drive. A cognitive-emotional resonance will typically activate a drive-value resonance, but a drive-value resonance can occur in the absence of a compatible external sensory cue.

## 5. Explainable Motor Representations

### 5.1. Computationally Complementary What and Where Stream Processes

The above examples all describe processes that take place in the ventral, or What, cortical stream. Only What stream representations for perception and cognition (Mishkin, 1982; Mishkin et al., 1983) are capable of resonating, and thus generating conscious representations, as examples of the general prediction that *all conscious states are resonant states* (Grossberg, 1980, 2017b). Criteria for a brain representation to be explainable are, however, weaker than those needed for it to become conscious. Accordingly, some properties of representations of the dorsal, or Where, cortical stream for spatial representation and action (Goodale and Milner, 1992) are explainable, even though they cannot support resonance or a conscious state.

This dichotomy between What and Where cortical representations is clarified by the properties summarized in Table 1, which illustrates the fact that many computations in these cortical streams are computationally complementary (Grossberg, 2000, 2013). As noted in Table 1, the What stream uses *excitatory matching*, and the *match learning* that occurs during a good enough excitatory match can create adaptive resonances (Figure 4) which learn recognition categories that solve the stability-plasticity dilemma. Match learning can occur when a bottom-up input pattern can sufficiently well match a top-down expectation to generate a resonant state (Figure 4) instead of triggering a memory search (Figures 3B,C). In contrast, the Where stream uses *inhibitory matching* to control reaching behaviors that are adaptively calibrated by *mismatch learning*. Mismatch learning can continually update motor maps and gains throughout life.

**Table 1:** The What ventral cortical stream and the Where dorsal cortical stream realize complementary computational properties. See the text for details [reprinted with permission from Grossberg (2009)].

**WHAT**	**WHERE**
Spatially-invariant object learning and recognition	Spatially-variant reaching and movement
Fast learning without catastrophic forgetting	Continually update sensory-motor maps and gains
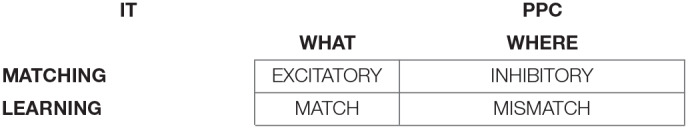	

Section 5.2 describes the VITE model of how arm movement trajectories are formed and executed. In particular, when an arm's *target position vector*—or the position where the hand wants to move—equals its *present position vector*—or the position where the hand is now—then the hand is where it wants to be, so stops moving. Correspondingly, the *difference vector* that is formed by subtracting the present position vector from the target position vector then equals zero. This kind of match cannot resonate. Hence, it cannot solve the stability-plasticity dilemma. As a result, the parameters that control arm movements can adjust throughout life to adapt to bodily development, exercise, and aging. As I will describe in Sections 5.3–5.5, this is a kind of mismatch learning, unlike the match learning that takes place in ART. I will also explain that it is unlike the kind of mismatch, or error-based, learning that takes place in back propagation and Deep Learning.

### 5.2. Vector Integration to Endpoint Computations Control Reaching Behaviors

Just as an attended pattern of critical features is explainable in the What cortical stream, a *difference vector* is explainable in the Where cortical stream, as are other vectors that are used for arm movement control. As explained below, observation of a difference vector is sufficient to determine the direction and distance of an impending reaching movement by an arm.

**Figure 12:**
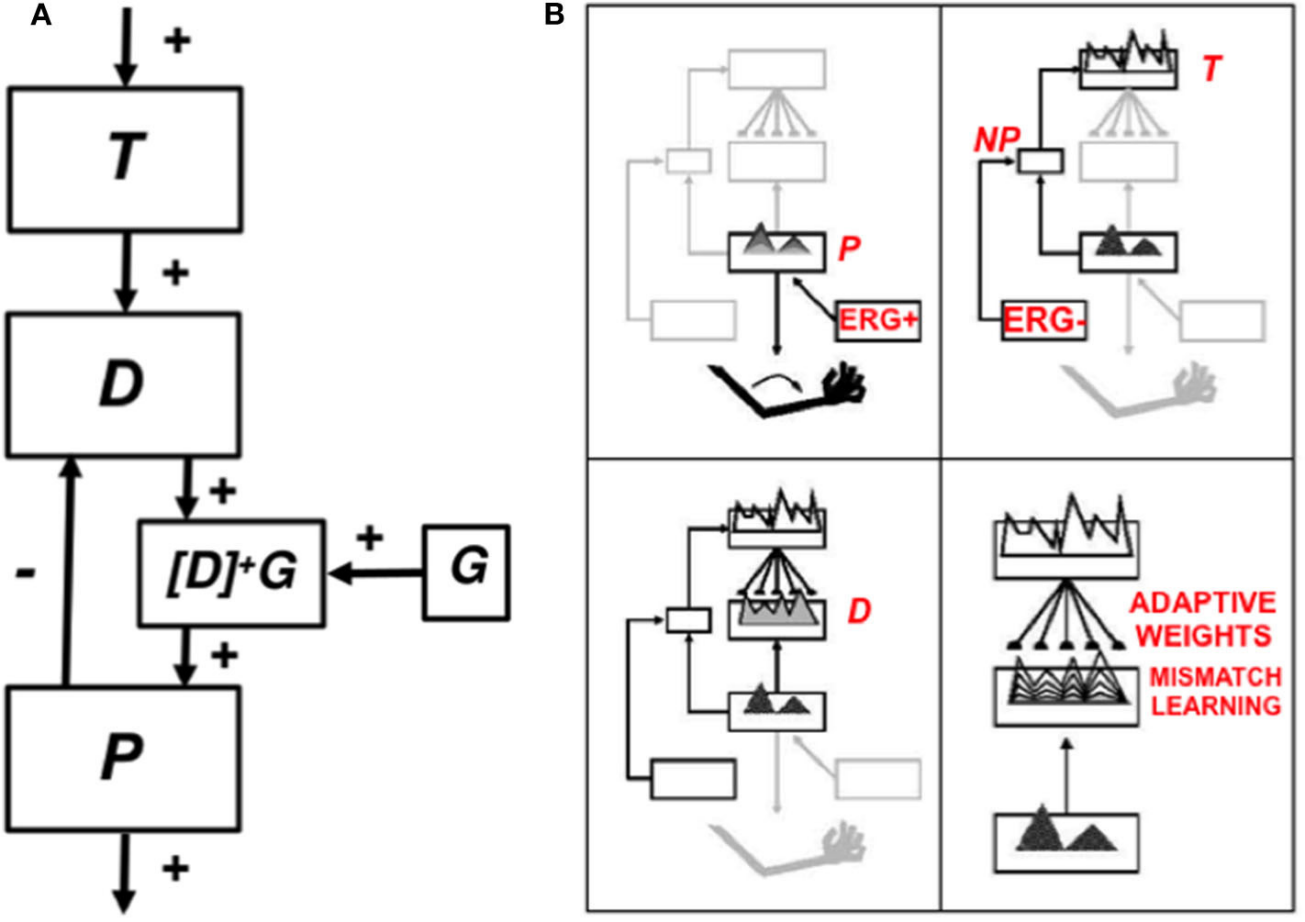
The Vector Integration to Endpoint, or VITE, model of Bullock and Grossberg (1988) realize the Three S's of arm movement control: Synergy, Synchrony, and Speed. See the text for details [adapted with permission from Bullock and Grossberg (1988)].

A difference vector is computed as part of the Vector Integration to Endpoint, or VITE, model of arm movement control (Figure 12A; Bullock and Grossberg, 1988, 1989). The VITE model clarifies how the Three S's of reaching—Synergy, Synchrony, and Speed—are achieved. To do this, the model computes a *target position vector* T, an outflow *present position vector* P, a *difference vector* D, and a volitional *GO signal* G. Variable T computes the position that the end of the hand/arm wants to go, P the position where it is now, D the direction and distance that it has to go to reach T, and G the motor energy needed to support this movement.

The first S is the flexible choice of a motor Synergy. Choosing T defines the collection, or Synergy, of muscle groups that will contract to carry out the arm movement to the desired target position. The second S controls the Synchronous performance of this arm movement. In other words, the muscles in the chosen Synergy contract together during the same time interval, even if they contract by different amounts. Finally, the third S enables the VITE model to cause the same arm movement trajectory to be executed at variable Speeds. Increasing the GO signal translates into a faster performance speed.

These properties follow from the fact that the GO signal G *multiplies* the difference vector D, or more precisely the thresholded difference vector [*D*]^+^ = *max*(*D*, 0), to form the product [*D*]^+^*G* in Figure 12A. Multiplication by G does not change the direction that D computes, so the same arm movement trajectory can be traversed in response to any positive value of G. The product [*D*]^+^*G* is integrated through time by P until P equals T. This property explains the name, VITE, of the model. When P equals T, the arm has attained the desired target position.

Multiplying by G ensures that all muscles in the Synergy get integrated with velocities that scale proportionally with the distances that they have to travel, so that contractions within the Synergy are Synchronous during the movement. Indeed, [*D*]^+^*G* is the *outflow velocity vector* of the circuit. Because [*D*]^+^*G* is integrated by P, increasing G increases the rate at which P approaches T, and thus the velocity of movement, while decreasing G causes slower movements.

All of these variables are explainable: Inspecting vector T discloses the movement's target position, P its current position, D the desired direction and distance of the movement, and [*D*]^+^*G* the movement's outflow speed.

**Figure 13:**
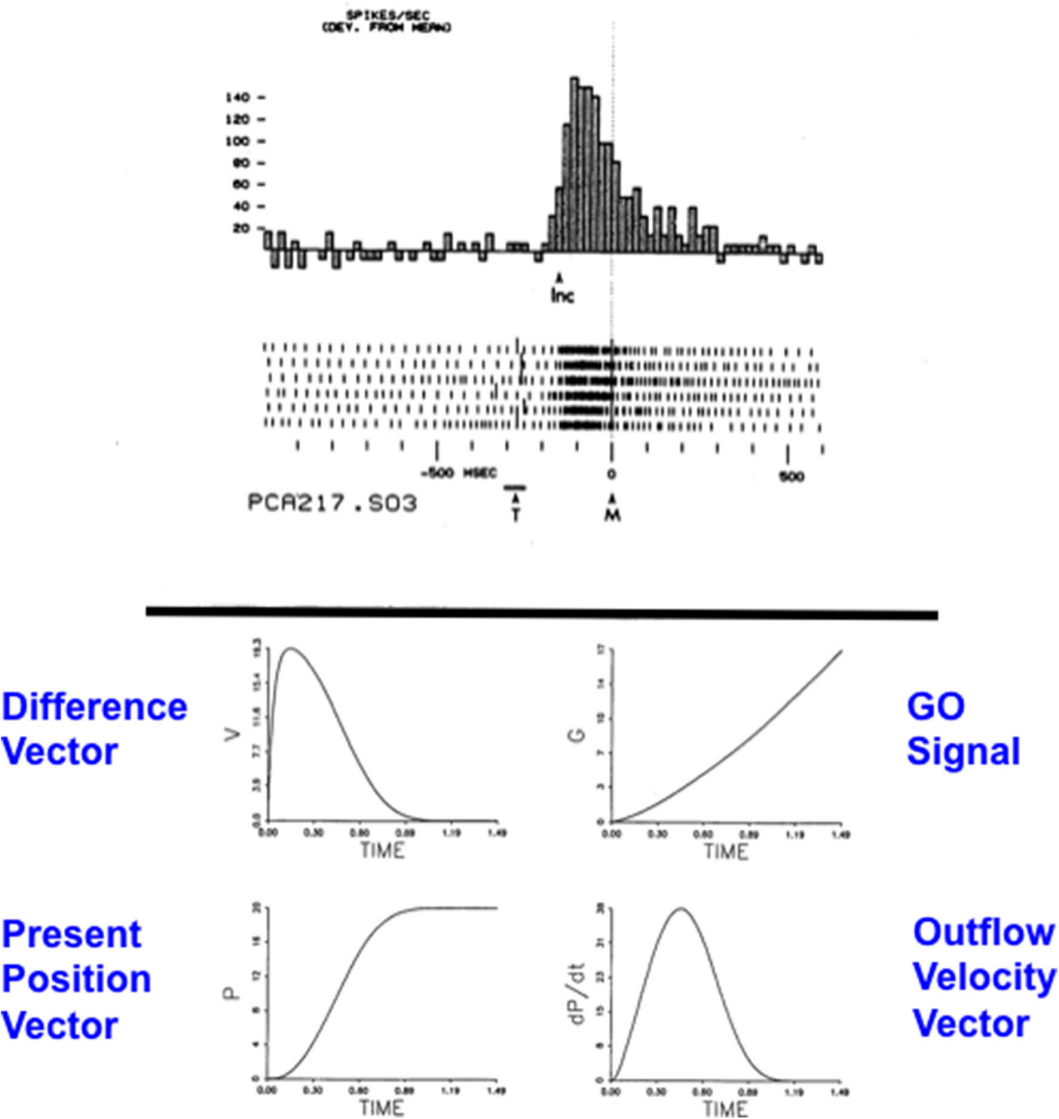
(Top half) Neurophysiological data of vector cell responses in motor cortex. (Bottom half) VITE model simulations of a simple arm movement in which the model's difference vector D simulates the data as an emergent property of network interactions [data of Georgopoulos et al. (1982) and Bullock and Grossberg (1988) are reproduced with permission. Figure as a whole is reprinted with permission from Grossberg (2020)].

An illustration of how STM variables and neurophysiological recordings contribute to explainable descriptions of brain data is given in Figure 13. The top half of Figure 13 summarizes neurophysiological data that was recorded by Georgopoulos et al. (1982) during a reaching movement of a monkey. The bottom half of Figure 13 shows how the VITE model simulates these data. In particular, the difference vector D of the VITE model closely matches the shape of the neurophysiological data. The VITE model has also been used to simulate many other psychophysical, anatomical, and neurophysiological data about arm movements, using the same set of parameters. It should be noted for readers who prefer spiking models to the rate model dynamics of VITE that any rate model whose cells obey the membrane equations of neurophysiological can be converted into a spiking model, without a change of key properties (Cao and Grossberg, 2012).

### 5.3. Mismatch Learning via Circular Reaction to Calibrate Spatial and Motor Networks

The signals used in VITE need to be calibrated throughout our lives by learning to ensure that they actually work the way that they are supposed to work. In particular, the inputs to D from T and from P come from different cell populations and are carried by different pathways. Signals to T often come from visual representations, whereas P is often coded in motor coordinates. If a VITE model is representing a postural state where T and P represent the same position, then D should equal zero. How are signals from T and P to D calibrated to assure this? This requires learning that matches the gains of the pathways. The Vector Associative Map, or VAM, model of Gaudiano and Grossberg (1991, 1992) generalizes VITE to explain how mismatch learning changes the gains in the T-to-D pathways until they match those in the P-to-D pathways.

The VAM model does this using a *circular reaction* that generates reactive movements which create a situation in which both T and P represent the same position in space. This happens when a person moves her arms around in space. I will explain in a moment what a circular reaction is and how it gets T and P to represent the same position in space. Given that this is so, if the VAM model were properly calibrated, the excitatory T-to-D and inhibitory P-to-D signals that input to D in response to the same positions at T and P would cancel, causing D to equal zero, since then the model has already moved the arm to where it wants it to be. If D is not zero under these circumstances, then the signals are not properly calibrated. VAM uses such non-zero D vectors as mismatch, or error, teaching signals that adaptively calibrate the T-to-D signals. As perfect calibration is achieved, D approaches zero, at which time mismatch learning self-terminates.

The concept of a circular reaction was introduced by Piaget (1945, 1951, 1952). As noted in Grossberg (2020), during a circular reaction, babies endogenously babble, or spontaneously generate, hand/arm movements to multiple positions around their bodies; see the Endogenous Random Generator, or ERG+, in the upper left panel of Figure 12B. When the ERG+ turns on, it activates P, and causes an arm movement. Random activities in the ERG+ generate random movements that sample the workspace. When the ERG+ shuts off, the movement ends, and a postural state begins. The offset of ERG+ triggers the onset of an opponent ERG- which opens a Now Print (NP) gate that allows P to be transmitted to T, as shown in the top right panel of Figure 12B. Then both T and P send signals to form the difference vector D, as in the lower left panel of Figure 12B.

If D equals zero, the network is properly calibrated. If not, then D acts as an error signal that drives adaptive weights, or LTM traces, in the T-to-D pathway to change until D does equal zero, as in the lower right panel of Figure 12B. Computer simulations in Gaudiano and Grossberg (1991, 1992) demonstrate how the model samples the work space during the critical period and learns the correct LTM traces with which to enable accurate later volitional movements to be made.

This kind of DV-mediated mismatch learning is just one of the kinds of mismatch learning in the Where cortical stream that is summarized in Table 1. Mismatch learning allows our spatial and movement control systems to adjust continually to our changing bodies through time. It is not the only kind of mismatch learning that occurs in these systems. A different kind of error-driven learning occurs in the hippocampus, cerebellum, and basal ganglia to control adaptively-timed motivated attention, motor control, and reinforcement learning. All of these brain regions seem to have similar circuits and underlying biochemical mechanisms, despite their very different behavioral functions.

### 5.4. Mismatch Learning Using Local Computations

Note that, unlike back propagation and Deep Learning, there is no non-local weight transport during the mismatch learning that occurs in Figure 12B. Rather, the teaching signals that drive this learning are vectors T that are locally transported from vectors P along pathways that form part of the network's physical anatomy, regulated by opponent ERG+ and ERG– control signals.

### 5.5. ART and VAM: A Self-Stabilizing Synthesis of Match and Mismatch Learning

There is also no collapse of recognition memory by catastrophic forgetting due to this kind of mismatch learning. As summarized in Table 1, ART explains how perceptual and cognitive processes in the What ventral processing stream use excitatory matching and match-based learning to solve the stability–plasticity dilemma so that perceptual and cognitive learning can occur quickly without causing catastrophic forgetting. Excitatory matching also supports sustained resonance, and thus sometimes conscious awareness, when a good enough match with object information occurs.

Match-based recognition learning also supports additional learning of recognition categories at higher cortical regions that are increasingly invariant under changes in an object's views, positions, and sizes when it is registered on our retinas. The 3D ARTSCAN Search model (Fazl et al., 2009; Cao et al., 2011; Foley et al., 2012; Chang et al., 2014; Grossberg et al., 2014) shows how such *invariant* category learning enables us to categorize and search the world without causing a combinatorial explosion of memories. However, positionally-invariant object category representations cannot, by themselves, manipulate objects at particular positions in space.

Complementary spatial and motor processes in the Where/How dorsal cortical processing stream can be used to manipulate objects in space using VAM inhibitory matching and mismatch learning to continually update spatial maps and sensory–motor gains as bodily parameters change through time. Their difference vector computations cannot support an adaptive resonance and thus do not solve the stability-plasticity dilemma.

Each type of matching and learning in Table 1 is thus insufficient to learn about the world and to effectively act upon it. But together they can. Perceptual and cognitive processes use excitatory matching and match-based learning to create self-stabilizing representations of objects and events that embody increasing expertise about the world. Complementary spatial and motor processes use inhibitory matching and mismatch learning to continually update spatial maps and sensory-motor gains to compensate for bodily changes throughout life. ART match learning provides a self-stabilizing perceptual and cognitive front end for conscious awareness and knowledge acquisition, and provides a stable platform upon which VAM mismatch learning enables our changing bodies to act effectively upon a changing world, without experiencing the catastrophic forgetting that afflicts back propagation and Deep Learning.

### 5.6. Motor Equivalent Reaching and Speaking: The DIRECT and DIVA Models

**Figure 14:**
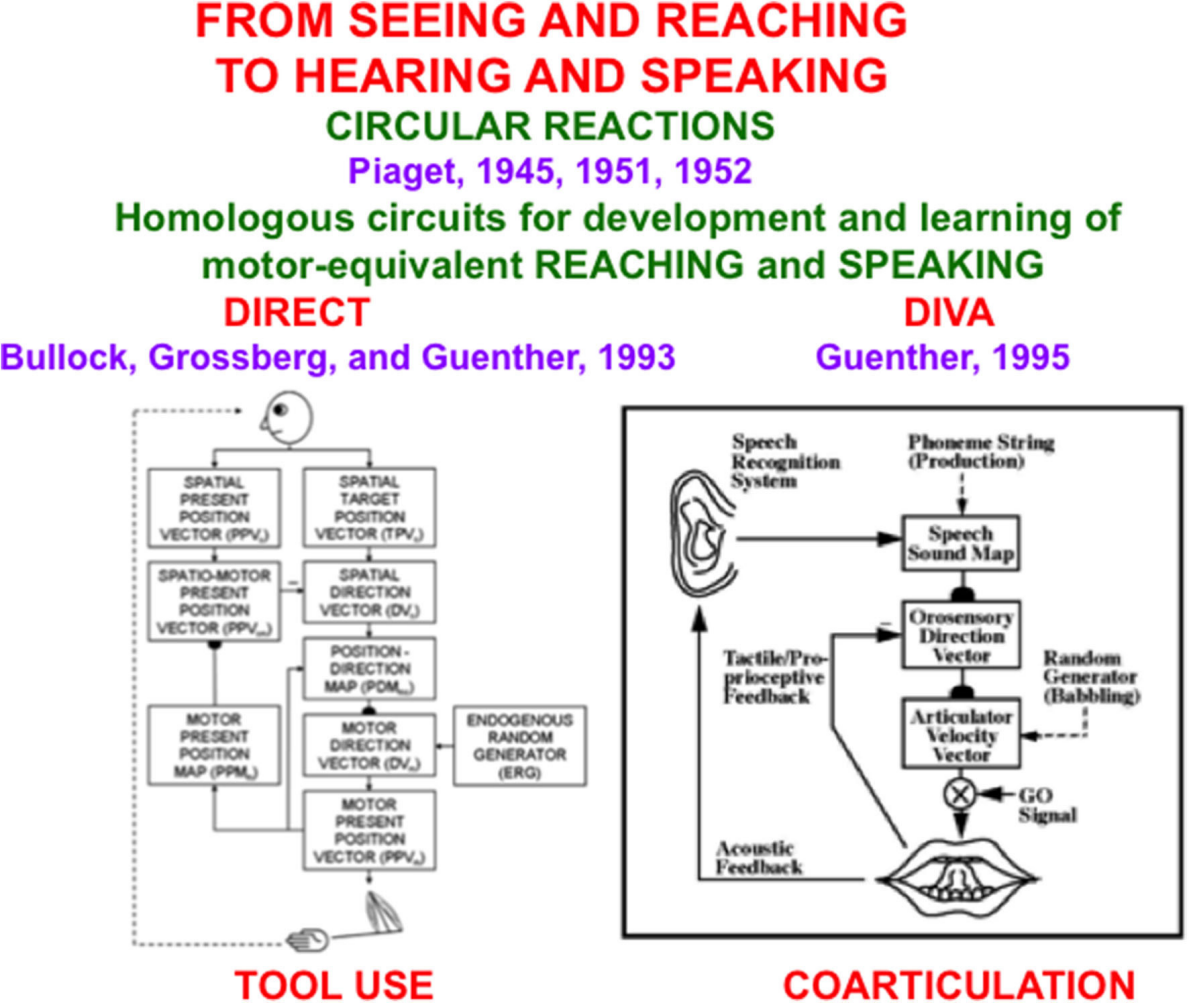
The DIRECT and DIVA models have homologous circuits to learn and control motor-equivalent reaching and speaking. Tool use and coarticulation are among the resulting useful motor-equivalent properties [reprinted with permission from Grossberg (2020)].

The VITE model has been substantially refined and developed over the years. These developments have shown, among other things, that models which explain how speaking may be controlled in response to auditory cues are homologs of VITE-like models for reaching in response to visual cues. The variant of VITE for which this is true is the DIRECT model of *motor-equivalent* reaching (Figure 14; Bullock et al., 1993), which includes the ability to reach a new target on the first try under visual guidance with either clamped joints or a tool. The speech homolog of DIRECT is the DIVA model (Figure 14), whose motor-equivalent speech includes the ability to compensate for coarticulation (Guenther, 1995; Guenther et al., 2006).

The property of motor equivalence enables spatially defined target objects to be reached using multiple arm movement trajectories. These multiple possibilities derive from the fact that a human arm is defined by components with a higher dimensionality than the dimension of a target object in space; e.g., a seven degree of freedom arm moving its hand along a desired path in three-dimensional space. Given that multiple trajectories are possible, how does a human or other primate choose among these possibilities to efficiently perform spatially defined reaching tasks?

The DIRECT model is able to fuse visual, spatial, and motor information to achieve this competence. An important part of this ability is to represent target information in space and to show how to transform it into motor commands; e.g., the *spatial* target position vector, spatial present position vector, and spatial direction vector in Figure 14 get converted into a *motor* direction vector and a motor present position vector that commands the arm's present position.

Another important part of this ability is that DIRECT learns these transformations between spatial and motor representations by using another circular reaction, albeit an *intermodal* circular reaction that links vision to action. As noted in Grossberg (2020), during a visual circular reaction, babies endogenously babble, or spontaneously generate, hand/arm movements to multiple positions around their bodies; see the Endogenous Random Generator in Figure 14. As a baby's hands move in front of her, her eyes automatically, or reactively, look at her moving hands. While her eyes are looking at her moving hands, the baby learns an associative map from its hand positions to the corresponding eye positions, *and* from eye positions to hand positions. Learning of the map between eye and hand in both directions constitutes the “circular” reaction. After map learning occurs, when a baby, child, or adult looks at a target position with its eyes, this eye position can use the learned associative map to activate a movement command to reach the corresponding position in space when a GO signal is activated (Figure 12A). Because our bodies continue to grow for many years as we develop from babies into children, teenagers, and adults, these maps need to continue updating themselves throughout life.

Computer simulations have been carried out in Bullock et al. (1993) of how DIRECT carries out reaches under various conditions, including how a reach is accurately made to a target in space on the first try, how the end of a tool touches the target position accurately on the first try without having to measure the length or the angle of the tool with respect to the hand, and how an accurate reach can be made on the first try with the elbow joint held fixed. A nearly accurate reach can also be made under memory guidance when the actor is blindfolded.

As can be seen by inspecting Figure 14, the DIRECT and DIVA models have homologous circuits to control arm movements and speech articulator movements, respectively. One evolutionary reason for this may be that speech articulators evolved from the same circuits that control chewing, and both motor-equivalent reaching and motor-equivalent chewing are integrated into a larger system for eating (MacNeilage, 1998).

DIVA models how learning and performance are initiated by an auditory circular reaction that occurs when babies endogenously babble simple sounds and hear the sounds that they create. When the motor commands that caused the sounds and the auditory representations of the heard sounds are simultaneously active in a baby's brain, it can learn a map between these auditory representations and the motor commands that produced them. After a sufficient amount of map learning occurs, a child can use the map to imitate sounds from adult speakers, and thereby begin to learn how to speak using increasingly complicated speech and language utterances, again under volitional control.

Both DIRECT and DIVA contain explainable representations, just as VITE does. Inspection of these representations as arms or speech articulators move “explains” how these competences are realized. For example, a clear correlation will exist between when an actor is looking and its spatial target position vector. A more detailed review of various of these model developments is found in Grossberg (2017b, 2020).

## 6. Explainable Autonomous Adaptive Intelligence: Hippocampus and Sovereign2

### 6.1. Balancing Reactive and Planned Movements During Learning of a Route to a Valued Goal

The article has thus far summarized examples of explainable representations that are computed by brains during perception, cognition, emotion, and action. In addition to noting that the LTM traces of fuzzy ARTMAP can be represented as fuzzy IF-THEN rules which can be used to explain the basis of categorization and prediction dynamics in this model, the article has focused upon the critical role that activity, or STM, variables play in representing and controlling brain processes. All of the summarized STM representations are explainable. By contrast, back propagation and Deep Learning compute only LTM traces, and no STM traces or dynamics.

These examples are just a subset of those that can be explained in biological neural models. A great many other examples can be found in the SOVEREIGN (Gnadt and Grossberg, 2008) and SOVEREIGN2 (Grossberg, 2019a) architectures, which embody capabilities for autonomous adaptive perception, cognition, emotion, and action in changing environments. SOVEREIGN was designed to serve as an autonomous neural system for incrementally learning planned action sequences to navigate toward a rewarded goal. The acronym SOVEREIGN summarizes this goal: Self-Organizing, Vision, Expectation, Recognition, Emotion, Intelligent, Goal-oriented Navigation.

SOVEREIGN illustrates how brains may, at several different organizational levels, regulate the balance between reactive and planned behaviors. Balancing between reactive and planned movement during navigation occurs during the transition between exploratory behaviors in novel environments and planned behaviors that are learned as a result of previous exploration. During initial exploration of a novel environment, many reactive movements may occur in response to unexpected or unfamiliar environmental cues (Leonard and McNaughton, 1990). These movements may initially appear to be locally random, as an animal orients toward and approaches many stimuli. As the surroundings become familiar to an animal, it learns to discriminate between objects likely to yield reward and those that yield punishment. More efficient routes to the goal are learned during this process. SOVEREIGN begins to model how *sequences* of such behaviors are released at appropriate times during autonomous navigation to realize valued goals.

Such approach-avoidance behavior is often learned via a circular reaction (Figure 14), in this case a perception-cognition-emotion-action cycle during which an action and its consequences elicit sensory cues that are associated with them. Rewards and punishments affect the likelihood that the same actions will be repeated in the future (Figure 11). If objects are not visible when navigation begins, multiple reactive exploratory movements may be needed to reach them. Eventually, these reactive exploratory behaviors are replaced by more efficient planned sequential trajectories within a familiar environment. One of the main accomplishments of SOVEREIGN is to explain how erratic reactive exploratory behaviors lead to learning of the most efficient routes whereby to acquire a valued goal, without losing the ability to balance reactive and planned behaviors so that planned behaviors can be carried out where appropriate, while still retaining the ability to react quickly to novel challenges. These capabilities were demonstrated in SOVEREIGN by simulations showing how an animal or animat could, using its control structure, learn and execute efficient routes to a valued goal in a virtual reality environment.

### 6.2. Difference Vectors During Navigation and Reaching

SOVEREIGN proposes how the circuit designs for spatial navigation are homologous to those that control reaching behaviors. In both cases, difference vectors are computed to determine the direction and distance of a movement (cf. Figure 12). During both navigation and reaching, in order to compute a difference vector D, both a target position vector T and a present position vector P first need to be computed. The target position T can be computed, for both arm movements and navigation, from visual information. Because an arm is attached to the body, its present position P can be directly computed using outflow movement commands that explicitly code the commanded arm position. In contrast, when a body moves with respect to the world, no such immediately available present position command is available. During navigation, the ability to compute a difference vector D between a target position and the present position of the body requires more elaborate brain machinery. When D is computed, it determines the direction and distance that the body needs to navigate to acquire the target.

Navigational difference vectors are explainable, just as arm movement difference vectors are.

### 6.3. Hippocampal Place Cells: Computing Present Position and Target Position

Hippocampal *place cells* provide information about present position during spatial navigation. They selectively fire when an animal is in a particular position within an environment (O'Keefe and Dostrovsky, 1971; O'Keefe and Nadel, 1978; Muller, 1996). The GridPlaceMap neural model proposes how entorhinal grid cells and hippocampal place cells are learned during real-time navigation in realistic environments (e.g., Grossberg and Pilly, 2014). Once our brains can compute a difference vector between present and desired bodily position, a volitional GO signal can move the body toward the desired goal object, just as in the case of an arm movement (Figure 12). During navigation, a GO signal can control movements with different gaits, such as walk or run in bipeds, and a walk, trot, pace, and gallop in quadrupeds, as the GO signal size increases (Pribe et al., 1997). In summary, both navigational movement in the world and movement of limbs with respect to the body use a difference vector computational strategy.

A considerable amount of additional machinery is needed to successfully navigate in the world. Successful navigation requires that humans and animals be able to solve basic problems of social cognition, such as how a student can learn a skill from a teacher whose behavior is observed in a different spatial location. The ability to share *joint attention* between actors in different locations is part of this competence. The CRIB (Circular Reactions for Imitative Behavior) model explains and simulates how this may be done (Grossberg and Vladusich, 2010).

For the moment, I will just note that “social” place cells in the hippocampus can fire in a bat as it observes another bat navigating a maze to reach a reward. In these experiments, the observer bat was motivated to do this so that it could subsequently navigate the same route to get the same reward. Under these circumstances, a social place cell can fire in the brain of the observing bat that corresponds to the position of the observed bat (Omer et al., 2018; Schafer and Schiller, 2018, 2020). The position of the observed bat can then function as a spatial target position vector (cf. Figure 14) to guide the navigation of the observer rat along the route.

These observations suggest a role for hippocampus, as part of its interactions with many other brain regions, in computing both present position vectors P and target position vectors T during spatial navigation, thereby enabling difference vectors D to be computed from them that can control navigational directions.

Various additional processes that have been modeled with a view toward achieving true adaptive autonomous intelligence in a mobile agent, whether biological or artificial, have been summarized in the SOVEREIGN and SOVEREIGN2 architectures (Grossberg, 2019a, 2020). These interactions among circuits to carry out aspects of perception, cognition, emotion, and action clarify how the “places” that are computed in brain regions like the hippocampus can become integrated into “social” and other higher forms of behavior. All of the STM representations in these architectures are explainable, in principle, using neurophysiological and functional neuroimaging methods.

## 7. Concluding Remarks

As summarized at the end of Section 1, this article outlines an explainable neural architecture for autonomous adaptive mobile intelligence. Each of the Sections 2–6 focuses on a different functional competence whereby biological brains, no less than artificial devices and robots, may be designed to achieve such autonomy. In addition to its clarifying effects on understanding brains and designing AI systems, achieving a computational understanding of autonomous adaptive intelligence may be expected during the coming century to have transformative effects on all aspects of society.

## Author Contributions

The author confirms being the sole contributor of this work and has approved it for publication.

## Conflict of Interest

The author declares that the research was conducted in the absence of any commercial or financial relationships that could be construed as a potential conflict of interest.
